# Nutritional, Anthropometric and Sociodemographic Factors Affecting Fatty Acids Profile of Pregnant Women’s Serum at Labour—Chemometric Studies

**DOI:** 10.3390/nu13092948

**Published:** 2021-08-25

**Authors:** Magdalena Broś-Konopielko, Agnieszka Białek, Luiza Oleszczuk-Modzelewska, Barbara Zaleśkiewicz, Anna Różańska-Walędziak, Krzysztof Czajkowski

**Affiliations:** 1 II Faculty and Clinic of Obstetrics and Gynaecology, Medical University of Warsaw, 02-091 Warsaw, Poland; mbros@wum.edu.pl (M.B.-K.); oleszczuk.luiza@gmail.com (L.O.-M.); barbara.zaleskiewicz@gmail.com (B.Z.); aniaroza@tlen.pl (A.R.-W.); jtckcac@gmail.com (K.C.); 2 Department of Biotechnology and Nutrigenomics, Institute of Genetics and Animal Biotechnology of the Polish Academy of Sciences, 05-552 Magdalenka, Poland

**Keywords:** new-born, nutrition, diet, fatty acids, pregnancy, developmental programming

## Abstract

Diet influences the health of pregnant women and their children in prenatal, postnatal and adult periods. GC-FID fatty acids profile analysis in maternal serum and a survey of dietary habits were performed in 161 pregnant patients from the II Faculty and Clinic of Obstetrics and Gynaecology of the Medical University of Warsaw. Their diet did not fulfil all nutritional recommendations regarding dietary fat sources. Olive and rapeseed oil were the most popular edible oils. High usage of sunflower oil as well as high consumption of butter were also observed, whereas fish and fish oil intake by pregnant women was low. A chemometric approach for nutritional data, connected with anthropometric, sociodemographic and biochemical parameters regarding mothers and newborns, was conducted for diet and its impact estimation. It revealed four clusters of patients with differing fatty acids profile, which resulted from differences in their dietary habits. Multiparous women to a lesser extent followed dietary recommendations, which resulted in deterioration of fatty acids profile and higher frequency of complications. Observed high usage of sunflower oil is disquieting due to its lower oxidative stability, whereas high butter consumption is beneficial due to conjugated linoleic acids supply. Pregnant women should also be encouraged to introduce fish and fish oil into their diet, as these products are rich sources of long chain polyunsaturated fatty acids (LC PUFA). Multiparous women should be given special medical care by medical providers (physicians, midwifes and dietitians) and growing attention from the government to diminish the risk of possible adverse effects affecting mother and child.

## 1. Introduction

Diet is a crucial environmental factor influencing the health status of the whole population. Especially, the quality of diet of pregnant women is of utmost importance, as nutritional demand of the mother and fetus increases. Diet influences not only mothers’ health but also is pivotal for their children health and development in prenatal, postnatal and adult periods. The quality of the maternal diet is directly associated with maternal health and wellbeing, pregnancy and fetal outcomes, as well as the risk of pregnancy complications [1]. The Developmental Origins of Health and Disease (DOHaD) (or developmental programming) hypothesis originates from the “fetal origins of adult health” hypothesis, which claims that most conditions (physiological or pathological) that occur in adulthood originate in fetal life. This hypothesis highlights the crucial importance of the fetal and early postnatal environment in shaping long-term health, and links parental nutritional status to metabolic traits in offspring [2]. Developmental adaptations to nutritional signals are a normal part of development in anticipation of future environment. Consequently, a specific genotype can generate a variety of different phenotypes depending on environmental cues (including nutritional) during critical periods of development, i.e., periods of developmental plasticity. It has become increasingly acknowledged that the prenatal period and early postnatal period are considered critical windows of plasticity for programming of health in adulthood [3].

Specific dietary recommendations in pregnancy are meant to fulfil all the requirements of a mother and a child, but on a global scale they differ in some points according to both eating tradition and nutritional status of the population. They concern macro- and micro-nutrients, with fat constituting 20–35% of daily energy intake [3]. Dietary fatty acid stored in body stores of the mother have a direct effect on fetal and infant fatty acid status, as during pregnancy they are transferred to the fetus through the placenta and in the postnatal period they continue to be provided through the maternal milk. Dietary fats provide energy for growth, but also supply essential fatty acid (EFA), precursors for long chain polyunsaturated fatty acids (LC PUFA), which are essential constituents of the membrane lipids that maintain cellular and organelle physicochemical properties and are important mediators of gene expression and eicosanoid production. EFA includes c9,c12 C18:2 (linoleic acid, LA), which is a precursor of n6 fatty acids family, and c9,c12,c15 C18:3 (α-linolenic acid, ALA), which is a precursor of the n3 fatty acids family. These two families of fatty acids have different functions and are not interchangeable, making dietary provision of both essential. Moreover, LA and ALA compete for the same enzymes, including two desaturases (Δ5-desaturase (FADS1) and Δ6-desaturase (FADS2)) and elongase, required for their conversion into LC PUFA [4], and produce varieties of metabolites with diverse physiological and pathological activities [5]. LC PUFAs are required for the development of the fetus and the neonate, e.g., visual and cognitive development and that of the immune and cardiovascular systems, and may also influence fetal and later child growth [6]. Peculiar changes in dietary fat quantity and quality, especially in the fatty acid composition as well as the structural organization of dietary lipids, influence vulnerability for later life non-communicable diseases, e.g., obesity and cancer [3,7]. Fetus and neonates are able to synthesize LC PUFA from EFA but the rate of synthesis is rather small (1–10%) [4], which is not enough to fulfil all the needs of the growing and developing organism. Free fatty acids in the maternal circulation are the major source of fatty acids for transport across the placenta. Placenta transfers maternal PUFA to the fetal circulation both through passive diffusion and through selective mechanisms targeting LC PUFA, especially c4,c7,c10,c13,c16,c19 C22:6 (docosahexaenoic acid, DHA) and c5,c8,c11,c14 C20:4 (arachidonic acid, AA) [5,6]. It is also a place of placental fatty acid metabolism, which exerts an important role in guiding pregnancy and fetal outcome [5]. 

There are many dietary recommendations concerning EFA and LC PUFA supply by pregnant women. Regarding EFA, there are only Australian recommendations. Daily ALA requirement for pregnant women is 1 g, and for lactating women 1.2 g a day. Daily LA requirement for pregnant women is 10 g, and for lactating women 12 g [8]. In Poland DHA requirement was defined in combination with c5,c8,c11,c14,c17 C20:5 (eicosapentaenoic acid, EPA). The Polish Paediatric Society’s [9] recommended dose of EPA + DHA is 1–1.5 g a day. It is considered that pregnant women, who consume small quantities of fish, ought to supply 0.5–0.6 g of DHA a day in their diet from the onset of pregnancy [10]. In complicated pregnancies threatened with premature delivery, supplementation ought to be even greater, amounting to 1 g DHA a day [10]. The upper limit of a maximum daily dose has not been determined, but research indicates that the supply of DHA up to 1.2 g and a total of 2.7 g of n3 PUFA are safe [11]. The total daily requirement for n3 LC PUFA (DHA/EPA/DPA) according to Australian recommendations for pregnant women is 0.110 g for women aged 14–18 years, 0.115 g for pregnant women over 19 years, and for lactating women 0.140–0.145 g [11]. The maximum daily dose defined in the above requirements for these groups of patients has been determined as 3 g a day [8]. 

Nutritional studies that focus on the relationship between diet and disease primarily rely on different survey research using numerous questionnaires, e.g., food frequency questionnaires (FFQs) or dietary self-report measures, because they are relatively inexpensive and easy to administer. Connection of those data with anthropometric and biochemical parameters is the most effective tool regarding diet and its impact estimation. 

For this reason, the first aim of this study was to evaluate the frequency of consumption of different food products, mainly dietary fat sources, as well as sociodemographic and anthropometric parameters concerning mother and child. The second objective was to measure the fatty acids profile of serum samples of pregnant women at the time of labour. Different techniques of multivariate analyses (such as cluster analysis or linear discriminant analyses) were used to disclose hidden dependencies among analysed variables and to confirm their usefulness in nutritional studies, which was the third aim of this study. As a novel feature of this study, a chemometric approach has been applied as an objective approach for evaluation and data interpretation to reveal even very subtle interactions among these diversified variables. 

## 2. Materials and Methods

### 2.1. Characteristic of Patients

This study was approved by the Bioethics Commission of the Medical University of Warsaw (KB 158/2010) and has been performed in accordance with the ethical standards laid down in the 1964 Declaration of Helsinki and its later amendments. It was conducted in the II Faculty and Clinic of Obstetrics and Gynaecology of the Medical University of Warsaw after receiving the conscious written consent of the patients of Anna Mazowiecka Clinical Hospital. Full-term pregnant women received the questionnaires on their nutritional status and habits before labour. A questionnaire was drawn up together with the Bromatology Department of the Faculty of Pharmacy with the Laboratory Medicine Division of the Warsaw Medical University (Appendix B), and enabled a detailed assessment of a pregnant women’s diet from the point of view of the consumption of various food products, mostly dietary sources of fat. The questions referred to the consumption statement (defined as “yes” or “no”) and frequency (defined as “never”, “once a two weeks”, “once a week”, “2–3 times a week”, “4–6 times a week”, “everyday”) of consuming the food products and their estimated quantity/mass per helping. 

The height and weight of the mothers were measured at the beginning of pregnancy and before the labour. Infants’ height, weight, head circumference and chest circumference were measured at birth. All measurements were performed by the qualified medical staff (midwives, physicians, obstetricians). They also filled in the three parts of questionnaire (Appendix B) concerning, e.g., chronic diseases before pregnancy (diseases of the kidneys, liver, thyroid, etc.) and diseases diagnosed during pregnancy (mainly hypertension, gestational diabetes, cholestasis and others). Anthropometric, clinical and pathological data for the studied patients are presented in Table A1 (Appendix A). A total of 161 women were included in the study. Serum samples were obtained from all of the pregnant women and completed questionnaires concerning diet were obtained from 135. Fatty acids profile was investigated for all of them and dietary characteristics were only investigated for the group of 135 cases. The descriptive analysis of the study population (cases) revealed that the mean age of the women in the study was 31.1 ± 4.5 years and the mean height was 167 ± 6 cm. The mean body weight of the investigated patients at the beginning of the pregnancy was 63.0 ± 11.8 kg, which resulted in mean BMI 22.6 ± 3.8 kg/m^2^. At the time of delivery the mean body weight was 77.0 ± 13.1 kg, which resulted in mean BMI 27.7 ± 4.1 kg/m^2^. Before pregnancy BMI of most of the women was in the healthy range (64.0%), whereas 49.1% were overweight and 19.3% developed first grade obesity. Most women (75.8%) had higher education, 27% having secondary education and only 5% vocational and 1.2% elementary education, respectively. Only one of the investigated patients declared tobacco smoking (0.6%) and none of them claimed alcohol usage during pregnancy. Correct course of pregnancy, which referred to uncomplicated pregnancy, was recorded in 64% of the women. 

Among the newborns there were slightly more girls, 87 (54%) than boys 74 (46%). Mean infant’s weight was 3464 ± 435 g, length—54.5 ± 2.9, head circumference—34.6 ± 1.5 cm, chest circumference—33.7 ± 1.8 cm and the mean Ponderal Index was 21.6 ± 2.7 kg/m^3^. The condition of the newborns according to the Apgar scale was determined as good for 100% after the 10th minute. The majority of newborns (80.7%) were healthy.

### 2.2. Fatty Acids Analysis

For the assessment of serum maternal fatty acids profile, 10 mL of maternal venous blood samples were collected at delivery. Whole blood was collected into a clot tube and centrifuged. Separated serum samples were stored at −80 °C until fatty acids were analysed.

Fatty acid analysis was made with gas chromatography (GC) using gas chromatograph (GC-17A gas chromatograph, Shimadzu, Kyoto, Japan) equipped with capillary column (BPX 70; 30 m × 0.25 mm i.d., film thickness 0.20 μm, SGE, Ringwood, Australia) and flame-ionization detector (FID). Helium (Multax) was the carrier gas. The initial oven temperature was 140 °C for 5 min, thereafter increased by 4 °C/min to 240 °C. The injector was heated to 250 °C and the detector to 270 °C. Fatty acids methyl esters (FAME) standards (Supelco 37 Component FAME Mix, Sigma, St. Louis, MO, USA) were used to identify and quantify the fatty acids percentage share in samples. 

The serum samples were thawed only once and three parallel samples of 100 μL were trans-esterificated according to the procedure of Bondia-Pons et al. [12] with minor modifications. The serum samples were hydrolyzed without prior lipid extraction by heating for 10 min with 2.5 mL sodium methoxide in methanol (0.5 mol/l) at 80 °C. Fatty acids were converted to methyl esters by heating with 2.5 mL of 14% boron trifluoride-methanol reagent at 80 °C for 3 min. FAME were isolated with hexane (2 × 0.5 mL) after adding 1.0 mL of saturated sodium chloride solution. Organic extracts were dried with anhydrous sodium sulphate and evaporated to dryness under a stream of nitrogen. FAME were diluted in 50 μL and stored at −20 °C until analysed. 1 μL of analytical sample was subjected into the column. Three parallel samples were prepared from each serum sample and results were expressed as mean percentage share of each individual fatty acid in the total fatty acid pool in the serum sample.

### 2.3. Statistical Analysis

For continuous variables, means and standard deviations were calculated. Categorical variables were described using frequencies and percentages. 

In order to better understand the data trends, fatty acids percentage shares were used as chemical descriptors to study possible discrimination of serum samples. Statistica 13.0 (StatSoft, Poland) was used for the chemometric analyses. Prior to this, the original data were transformed into natural logarithms and then standardized. 

Similarity analysis was performed by grouping of features and objects to prepare a heat map. Cluster analysis (CA) was performed to determine the similarity of the samples’ examined variables (fatty acids). Hence, CA was also performed to determine the similarity of examined serum samples described by the set of variables (fatty acids percentage share). These analyses were carried out using an agglomeration method. The Euclidean distance was used as the distance determination method and the Ward method was used as the agglomeration method. The application of less restrictive Sneath’s criterion (66%) was used for dendrograms analyses and cluster distinguishing. For variables for which the variance homogeneity assumptions were fulfilled, one-way analysis of variance (ANOVA) with post hoc Tukey’s test was used to determine the differences among existing clusters of serum samples. If the assumptions of the analysis of variance were not met, the non-parametric Kruskal-Wallis test (marked * in Table 1), which is a non-parametric equivalent of one-way ANOVA, with post hoc multiple comparison test was used to assess differences among clusters of serum samples. The accepted significance level was established at *p* < 0.05. 

Moreover, in order to obtain appropriate classification rules for serum samples into distinguished clusters Sk1-Sk4, a linear discriminant analysis (LDA) for fatty acids profile was performed. Relevant discriminant functions were calculated via a stepwise progressive method, with the adopted tolerance value of 1 − R^2^ = 0.01 to optimize LDA. 

LDA was also performed for variables concerning the frequency of dietary products intake. Variables with zero variance have been a priori excluded from the model (oils: corn, coconut, peanut, hemp, pumpkin and eel). Relevant discriminant functions were calculated in a stepwise progressive method, with the adopted tolerance value of 1 − R^2^ = 0.01 to optimize LDA.

## 3. Results

### 3.1. Diet Analysis

Completed questionnaires obtained from the patients were used for analysis of diet during pregnancy. Main findings from diet analysis are presented below whereas detailed data are presented in Appendix A (Table A2, Table A3, Table A4, Table A5, Table A6, Table A7 and Table A8). 

Olive oil and rapeseed oil were the most popular edible oils among pregnant women. 5.0% of patients incorporated olive oil in their diet daily and only 26.1% of them did not use it at all. Similarly, 6.8% of patients consumed rapeseed oil every day, but in the diet of 46.6% no rapeseed oil was used. Sunflower oil was also frequently eaten (3.7% consumed sunflower oil daily). Only one patient consumed flaxseed oil daily and almost 80% all patients never introduced linseed oil into their diet. Similarly, about 80% of investigated patients did not consume any corn, coconut, grapeseed, sesame, soybean, peanut, hemp or pumpkin oils (Table A2).

Among dietary fats, butter was consumed by 36.6% of patients daily and only about 30% of all patients entirely eliminated butter from their diet. Soft margarine and butter-margarine mix were consumed each day by a much lower percentage of patients and about 80% of patients eliminated these spreadable fats from their diet. Similarly, about 80% of patients completely excluded lard from their diet. Only 55.3% of all investigated women never used margarine. Mayonnaise was quite frequently consumed (most of the investigated patients (21.1%) ate it once every two weeks, whereas only 42.2% never used it in their diet (Table A3).

None of the investigated patients consumed fish every day (Table A4). Most of the patients ate fish once every two weeks. Only 1.2% of the investigated women ate herrings 4–6 times a week. 2–3 times a week salmon, tuna mackerel and cod were chosen by 5.6%, 2.5%, 1.2% and 0.6% of patients, respectively. Frequency of trout, sprat and pollock consumption did not exceed 1 serving per week for 1.9%, 1.2% and 0.6% of patients, respectively. Only 0.6% and 1.9% of patients consumed sardine and halibut once every two weeks, respectively.

Nuts seem quite popular in the diet of investigated patients (Table A5). Almonds seem to be the most popular, as they were daily consumed by 3.9% of all participants. In addition, 2.9% of patients ate hazelnuts and 0.6% of patients ate peanuts daily. About 50% of women totally excluded hazelnuts, walnuts and almonds from their diet and, in the case of cashews, pistachios and peanuts, those percentages were higher.

Most of the patients included eggs in their diet once a week (34.2%) or 2–3 times a week (29.2%) and for 6.8% of investigated women eggs were an indispensable element of their daily diet. Only 3.7% of patients totally excluded eggs from their diet (Table A6).

Data concerning other food products (flakes, cereals, bread, green vegetables and other) are given in Table A7 and Table A8. In brief, only 3.1% of patients decided to include fish oil in their diet whereas potato fries and chips were present in the diet of 31.1% and 18.6%, respectively. On the other hand, investigated patients frequently used different dietary supplements. Over 62% used vitamin supplements and 40.4% of used omega supplements. 

### 3.2. Serum Fatty Acids Profile of Investigated Patients

In our experiment we analysed 24 fatty acids, including ten saturated fatty acids (SFA), six monounsaturated fatty acids (MUFA) and eight polyunsaturated fatty acids (PUFA). These were the fatty acids present in serum samples in the highest amounts, which were able to be identified and quantified with the commonly used GC-FID technique. Palmitic (C16:0), oleic (c9 C18:1, OL), linoleic (c9,c12 C18:2, LA), stearic (C18:0) and arachidonic (c5,c8,c11,c14 C20:4, AA) were found to be the main fatty acids in the serum of patients in the study. Overall shares of identified and quantified SFA, MUFA and PUFA were comparable (Table 1). Other fatty acids present in serum samples, which were not identified, were present in much smaller amounts.

#### 3.2.1. Cluster Analysis

**Figure A1:**
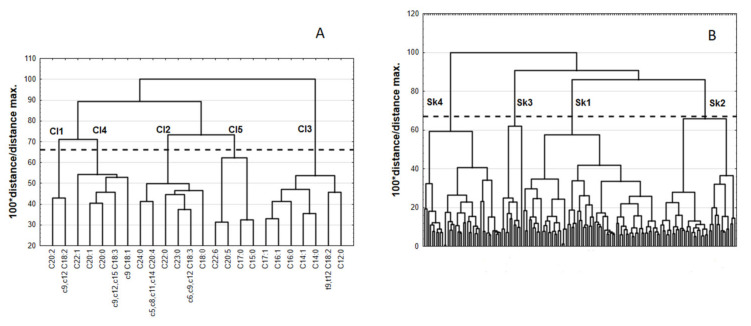
Dendrograms of similarities among fatty acids (**A**) and fatty acids profile of serum samples (**B**). Sk1–Sk4—clusters of serum samples revealed in cluster analysis.

Two cluster analyses were performed to reveal hidden similarities of the investigated objects. The first cluster analysis was performed to determine the similarity of the examined variables (fatty acids). The second cluster analysis was performed to determine the similarity of examined serum samples described by the fatty acids percentage share. This attempt also enabled the heat map preparation in the next step of data evaluation. The results of CA are presented as dendrograms in Figure A1a,b. The application of the less rigorous Sneath’s criterion (66%) to the dendrogram analysis distinguished five clusters (Cl1–Cl5) that grouped the examined fatty acids (Figure A1a). The first cluster (Cl1) included LA and C20:2, whereas C18:0, c6,c9,c12 C18:3 (GLA), AA, C22:0, C23:0 and C24:0 were incorporated into second cluster (Cl2). C12:0, C14:0, C14:1, C16:0, C16:1, C17:1 and t9,t12 C18:2 created the third cluster (Cl3) and the fourth cluster (Cl4) OL, ALA, C20:0, C20:1 and C22:1 were included. The fifth cluster (Cl5) consisted of four fatty acids: C15:0, C17:0, c5,c8,c11,c14,c17 C20:5 (EPA) and DHA. The dendrogram of similarities in fatty acids share in serum samples revealed four clusters (Sk1-Sk4), due to the application of the less rigorous Sneath’s criterion (66%) (Figure A1b). Share of fatty acids in serum differed significantly among samples allocated to revealed clusters (Table 1). The highest content of both fatty acids of Cl1 was quantified in serum samples of Sk1 and its content in other clusters was significantly absent. The main fatty acid of Cl2 was C18:0, which, in a significantly higher amount than in others, was detected in samples creating Sk2. Its high share was also observed in serum of Sk1 and it significantly exceeded C18:0 share in samples of Sk4. As far as other SFA, content of 24:0 did not differ among clusters whereas the lowest content of C22:0 and C23:0 was revealed in Sk4. Of PUFA included in Cl2, the content of GLA in Sk3 predominated in other clusters. However, in the case of AA, its highest levels were detected in serum samples of Sk2 and they differed significantly from amounts revealed in Sk1 and Sk4. All fatty acids allocated to Cl3 predominated in serum samples of Sk1. OL was the main fatty acid creating Cl4 and its highest share was observed in samples creating Sk4. It exceeded c9 C18:1 levels in Sk1 and Sk2. PUFA in Cl4—ALA predominated in Sk3, significantly higher than its shares in other clusters. Sk3 was also distinguished by the highest levels of C20:0 and C20:1, and in Sk4 the highest levels of C22:1 were observed. Two SFAs included in Cl5 were detected in similar amounts and their lowest share was observed in serum samples of Sk4, whereas two LC PUFA, EPA and DHA, predominated in serum samples of Sk2 with slightly lower levels detected in samples of Sk1. They significantly exceeded EPA and DHA share in serum of Sk4. 

The predominating content of individual medium-chain SFA in Sk1 resulted in the highest overall level of SFA in serum samples of this cluster (Table 1). MUFA levels were comparable in revealed clusters except for Sk2, in which their content was significantly lower. This was compensated for by the highest level of PUFA, especially n6 PUFA in serum.

#### 3.2.2. Grouping of Features and Objects—Similarity Analysis

**Figure 1:**
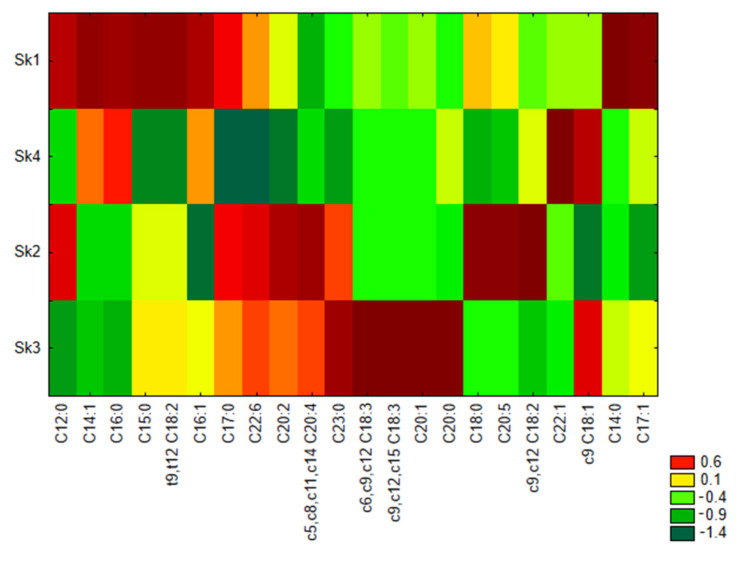
Heat maps of fatty acids mean percentage share in serum samples of distinguished clusters. Sk1–Sk4—clusters of serum samples revealed in cluster analysis.

Similarity analysis was also performed by the method of grouping of features and objects for fatty acids differing significantly among clusters revealed in CA. This was applied to prepare a heat map (Figure 1) and clearly shows that serum samples of Sk1 were characterized by the highest share of medium-chain SFA (C12:0, C14:0, C15:0, C16:0 and C17:0) and t9,t12 C18:2, as well as the lowest share of AA. In samples included in Sk2, the highest share of only one SFA was detected. In Sk2 samples the highest levels of medium and long-chain PUFA (LA, C20:2, AA, EPA and DHA) were observed, whereas medium-chain MUFA (C16:1, C17:1 and OL) were present in the lowest amounts. For Sk3 the highest share of two PUFAs (GLA and ALA) and C20:0, C20:1 and C23:0, as well as the lowest amounts of C12:0, C16:0 and LA, were distinctive. In serum samples included in Sk4, the highest share of C22:1 and OL as well as the lowest share of C17:0 and DHA were observed.

#### 3.2.3. Linear Discriminant Analysis

In the next step, LDA was used to obtain appropriate classification rules for classification of the examined serum samples into distinct clusters, Sk1-Sk4. Relevant discriminant functions were calculated in a stepwise progressive method. Percentage share of 24 fatty acids, which were detected in all examined serum samples, were included in the LDA1 model. In the analysis, 21 variables were included in the final model, and 13 of them (C20:1, C17:1, GLA, C18:0, C22:1, OL, C23:0, C16:1, EPA, ALA, C15:0, C20:2 and LA) were significant. All of these made a comparable contribution to overall discrimination. Applied canonical analysis distinguished three statistically significant (*p* < 0.0001) discriminant functions (DF). DF1 is the most significant function, as it explains 62.4% of discriminatory power; DF2 explains 23.1% of discriminatory power whereas DF3 explains 14.5% of discriminatory power, respectively (Table A9).

**Figure 2:**
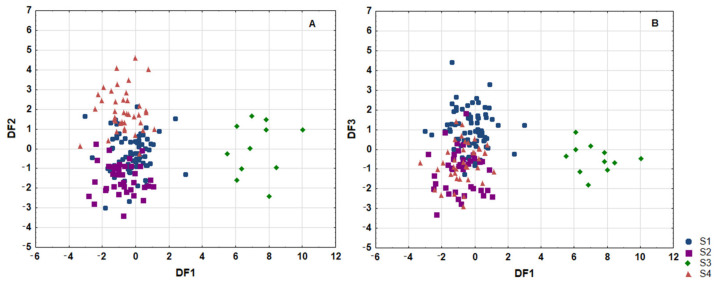
Scatterplot of canonical values for functions DF1 and DF2 (**a**) and DF1 and DF3 (**b**). Sk1–Sk4—distinguished clusters.

Analysis of canonical mean variables indicated that DF1 had the greatest impact on the distinction of Sk3 serum samples from others, DF2 seemed to distinguish Sk4 serum samples from Sk2 serum samples, whereas DF3 seemed to distinguish Sk1 serum samples mostly from Sk2 (Table A9). Graph analysis confirms the suggestion provided by the values of average canonic variables (Figure 2a,b).

The calculated classification matrix indicated that average classification efficiency based on the calculated functions was 88.8% (Table 2). For individual groups these coefficients were as follows: 100% for serum samples of Sk3, 92.5% for samples of Sk2, 87.3% for samples classified to cluster Sk1, and 84.6% for serum samples of Sk4, respectively.

### 3.3. Connection of Fatty Acids Profile and Diet

To verify, if nutrition is related to the fatty acids profile of serum and which products are of great importance for fatty acids profiling, LDA was performed to obtain appropriate classification rules for examined samples. Only those patients who provided data concerning their diet were included in the study. Variables concerning the frequency of food products consumption were included in the model. However, variables with zero variance have been a priori excluded from the study (oils: corn, coconut, peanut, hemp, pumpkin and eel). Relevant discriminant functions were calculated in a stepwise progressive method. In the analysis 19 variables were included in the final LDA2 model, and eight (wheat bread, almonds, butter, whole wheat bread, butter-margarine mix, omega-3 supplementation, salmon and sunflower oil) were significant. All of these made a comparable contribution to overall discrimination. Applied canonical analysis distinguished three discriminant functions (DF), but only two were statistically significant (*p* < 0.0001). DF1 is the most significant function, as it explains 49.7% of discriminatory power, whereas DF2 explains 34.1% (Table A10).

**Figure 3:**
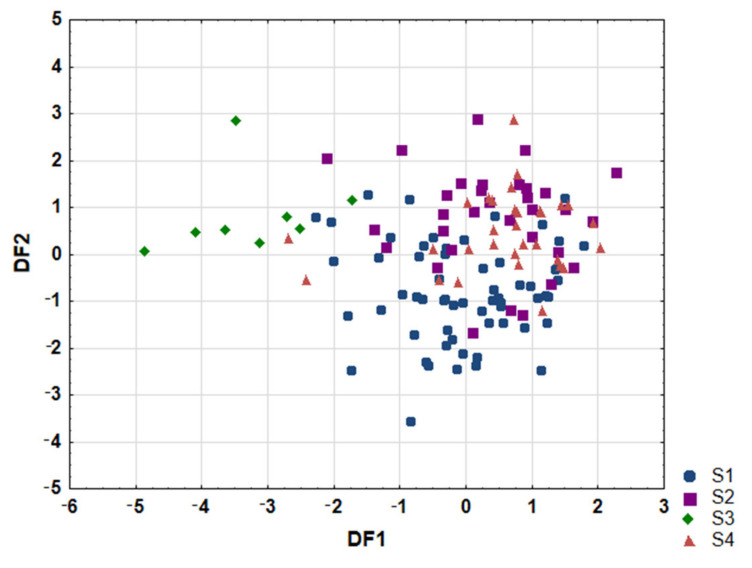
Scatterplot of canonical values for functions DF1 and DF2. Sk1-Sk4—distinguished clusters.

Analysis of canonical mean variables indicated that DF1 had the greatest impact on the distinction of Sk3 samples from others, whereas DF2 seemed to distinguish Sk1 samples from others. No pronounced distinguishing of Sk2 and Sk4 has been achieved (Table A10). Graph analysis confirms the suggestion provided by the values of average canonic variables (Figure 3).

The calculated classification matrix indicated that average classification efficiency based on the calculated functions was 71.8% (Table 3). For individual groups these coefficients were as follows: 87.5% for samples of Sk3, 80.5% for samples of Sk1, 64.5% for samples classified to cluster Sk4, and 59.4% for samples of Sk2.

Patients classified to Sk3 seem to be the most effectively separated from others. Their diet seems very monotonous (Table A2, Table A3, Table A4, Table A5, Table A6, Table A7 and Table A8). As many as 45.5% consumed wheat bread daily, whereas whole wheat bread and rye bread were much less popular, as 63.6% never ate both. However, cornflakes were consumed daily by 18.2% of Sk3 women. 63.6% of Sk3 patients included butter in their meals every day. On the other hand, butter-margarine mix and lard were not popular, as well as chips, which usually include palm oil. Their diet was poor in edible oils, and both sunflower oil and grapeseed oil were totally excluded from the diet of over 70% of these patients. Diet of Sk3 patients was also poor in fish, as most of them never chose fish and single women only occasionally (once a week or once every two weeks) included salmon, cod or herring in their diet. None of the Sk3 patients ate almonds or walnuts each day. They also totally excluded tofu from their meals. However, both vitamin supplementation and omega-3 supplementation were very popular among Sk3 patients, as 72.7% used these dietary supplements.

As far as Sk1 patients are concerned, their diet seems more multifarious. Most of them totally excluded wheat bread in favor of rye bread and whole wheat bread, which were consumed daily by 24.2% and 19.9% of Sk1 patients, respectively. Butter was the main fat in their diet and 36.6% used it daily, whereas butter-margarine mix and lard were completely excluded from their diet by about 80%. Women in the Sk1 cluster willingly consumed almonds and walnuts, as well tofu, to diversify their meals. The diet of Sk1 patients was characterized also by higher intake of fish and salmon, and cod and herrings were consumed by some of these patients even 2–3 times a week. Dietary supplementation with vitamins was applied by 62.1% of Sk1 patients whereas 40.4% used omega-3 supplements.

### 3.4. Characteristic Features of Revealed Clusters

Taking into account observed differences in fatty acids profile and in diet, we searched for the anthropometric and sociodemographic factors, which may be responsible for Sk3 patients’ separation. Among Sk3 patients only four did not develop diseases in pregnancy whereas 63.6% suffered from different diseases (Table 4). In three of these, gestational diabetes type 1 (GDMG1) appeared, and two suffered from cholestasis (in one, accompanied by chronic hypertension PPH without proteinuria), which should be connected with introducing specific dietetic interventions. Moreover, one patient developed isolated proteinuria and one patient suffered from intrauterine infection. What is really symptomatic is the fact, that for most Sk3 patients it was the second (54.5%) or third (18.2%) pregnancy. More patients from Sk3 cluster (27.3%) were overweight before pregnancy than in other clusters. 54.5% of Sk3 patients had a caesarean section, but only for two of them was this planned, whereas for others it resulted from no progress from the threat of intrauterine asphyxia. Additionally, six of eleven newborns of Sk3 mothers developed some developmental disorders.

## 4. Discussion

Pregnancy is a period of intensive changes in a woman’s body. It is recommended that during pregnancy some nutrients should be supplied in increased quantities while others should be entirely eliminated [13]. PUFAs are particularly important and an essential element of a pregnant woman’s diet; for instance, increased supply of n3 fatty acids results in slightly longer pregnancy duration, higher birth weight, reduction in the risk of premature birth and better mental development during the first years of life [14,15,16,17]. Data on the consumption of different dietary food products as dietary sources of fat, as well as data concerning fatty acids, especially PUFA intake by pregnant women in Poland, are lacking.

In the present study, based on original questionnaires, the frequency of intake of different food products, mainly rich sources of fats, was established. Edible oils, dietary fats and fish and seafood consumption were evaluated as main sources of PUFA. 

Olive and rapeseed oil were the most popular edible oils among investigated pregnant women, which seems positive concerning their fatty acids profile. These are rich dietary sources of MUFA, especially OL of high oxidative stability and beneficial health influence. A similar tendency concerning olive oil was observed by Aparicio et al. [18]. High usage of sunflower oil, also established in the present study, especially for frying, seems disquieting due to its lower oxidative stability resulting from high LA content, etc. [19]. High consumption of butter, which was the most popular spreadable dietary fat, seems beneficial due to the fact that butter is a rich source of conjugated linoleic acids (CLA) in the diet of mothers. Our previous results clearly indicated that proper CLA supply in the maternal diet may beneficially influence children’s health also in adulthood [1,20,21]. Maternal serum fatty acids profile generally was similar to that revealed in studies by other authors [18,22,23,24].

The human body does not synthesize EFA [25]; however, it is capable of synthesizing DHA and EPA from ALA, but the production of a woman’s owns body is insufficient to satisfy pregnancy requirements [10]. Principal dietary sources of n3 PUFA are fish and seafood and most dietary guidelines recommend two–three seafood meals per week. In the present study, frequency of fish consumption by examined pregnant women was very low and seafood was almost totally excluded from their diet, which resulted in a rather low estimated dietary intake [26] and low levels of LC PUFA in the serum of investigated patients. That amount of fish and seafood in the diet of pregnant women does not satisfy the daily requirement for EPA and DHA. Low fish consumption by pregnant women does not only apply to the Polish population. The Environmental Protection Agency and the Food and Drug Administration conducted studies which assessed fish consumption by American pregnant women. 50% of pregnant women ate fewer than 2 ounces a week, far less than the amount recommended [27]. Meanwhile, studies by Aparicio et al. confirmed, that fish and seafood consumption increased EPA concentration and reduced n6/n3 and AA/EPA values in the first and third trimesters, whereas its consumption increased DHA concentration only in the first trimester in pregnant Spanish women [18]. 

Low fish intake by Polish pregnant women may be explained by habitual tendencies. General fish consumption in Poland is low. In the studies of food product consumption among the Polish adult population, Sygnowska et al. [28] demonstrated that fish intake among women as well as men was significantly lower than recommended and amounted to 15 g a day and 16 g a day, respectively, instead of the recommended 30 g a day and 35 g a day, respectively. In another study many women reported decreasing intake of both cooked fish and meats and alternatives; these changes are contrary to recommendations [26]. 

However, fish and seafood consumption is also combined with some risk as fish is a source of methylmercury (MeHg), which is neurotoxic, and even mildly elevated exposure during gestation can damage the developing brain [29]. Because of this, dietary recommendations for pregnant women concerning fish and seafood should not only persuade women to eat fish more often, but also guide them to choose varieties with more n3 PUFA and less MeHg. However, two systematic reviews utilizing methodologies detailed by the Dietary Guidelines for Americans Scientific Advisory Committee 2020–2025, after reviewing 44 publications on 106,237 mother-offspring pairs and 25,960 children, composed two conclusion statements, that (i) consumption of a wide range of amounts and types of commercially available seafood during pregnancy is associated with improved neurocognitive development of offspring as compared to eating no seafood and (ii) that consumption of >4 oz/week and preferably >12 oz/week of seafood during childhood has beneficial associations with neurocognitive outcomes [30]. 

Application of omega 3 dietary supplements, which were used by 40.4% of investigated patients, has also been proposed as an efficient way of providing n3 LC PUFA. Numerous studies have indicated that supplementation with DHA preparations increased the concentration of maternal DHA in erythrocyte phospholipids, serum and breast milk, and umbilical blood [31]. An advantage of dietary supplements made from algae is the absence of methylmercury contamination [31]. Studies by Jackson et al. [32] clearly showed that every increase in fish intake increased the omega-3 index by 0.50–0.65% (*p* < 0.0001), whereas taking an EPA + DHA supplement increased the omega-3 index by 2.2% (*p* < 0.0001), which confirms that supplementation with omega-3 dietary supplements is effective. They proposed consumption of at least three fish servings per week plus taking an EPA + DHA supplement to provide optimal LC PUFA intake. Kouba et al. observed that dietary supplementation of mares with marine-derived DHA or EPA/DHA during late gestation not only altered the fatty acids profile in plasma of mothers, resulting in a greater concentration of EPA and DHA, but also resulted in n3 LC PUFA transfer to the blood of dams [33]. However, positive dietary changes concerning fish consumption, which are required in order to prevent these deficiencies and provide an adequate intake to the child, may be difficult to make as (i) recommendations are complicated (women are recommended to eat fish but to avoid mercury-containing fish) and (ii) physiological symptoms of pregnancy such as nausea and aversions reduce the intake of some recommended foods [34]. 

During early pregnancy, LC PUFA derived from both the maternal diet and maternal metabolism are stored in maternal adipose tissue. During late pregnancy, enhanced lipid catabolism as a consequence of the insulin-resistant condition causes the development of maternal hyper-lipidaemia, which plays a key role in the availability of LC PUFA to the fetus [5]. In the first trimester, DHA is involved in the placentation process by stimulating tube formation [35]. The placental vascular network is essential for the growth and maintenance of the developing embryo, and LC PUFA of n3 and n6 families are directly or indirectly involved in angiogenesis. Metabolites of n3 LC PUFA attenuate excess vascularization, whereas the n6 LC PUFA have a stimulatory or neutral effect on angiogenic processes [5]. Alterations in LC PUFA metabolites result in inadequate spiral artery remodeling or placental angiogenesis. These structural and functional deficiencies of placenta increase the risk of pregnancy complications, such as preeclampsia, gestational diabetes mellitus and intrauterine growth restriction, and results in adverse birth outcomes [35].

LC PUFAs, especially DHA, play a pivotal role in the development of the central nervous system, visual acuity, and cognitive functions. This depends on their involvement in maintaining membrane fluidity, impulse propagation, synaptic transmission, and functioning as a cytosolic signal-transducing factors for various types of gene expression during the critical period of brain development, which seems to be the last trimester and first few months after birth. The highest accumulation of DHA by the fetus takes place during the third trimester of pregnancy and during the first years of life. DHA is accumulated mainly in the brain and retina [36]. Fetal brain growth is at its peak velocity during the last trimester and the first few months after birth, which makes the third-trimester fetus and new-born baby particularly vulnerable to LC PUFA deficits. Although it is possible to produce DHA from ALA, the ability to convert ALA to n3 LC PUFA depends on gene polymorphism (*FADS1* and *FADS2*) for Δ-5 and Δ-6 desaturase and [37] such conversion is insufficient for the requirements of a developing pregnancy [10]. Because of this, lack of an adequate supply of DHA in a pregnant woman’s diet may result in irreversible changes, and DHA deficiencies occurring during gestation and soon after birth cannot be fully corrected later in life [5].

Preconception maternal excessive body weight is associated with greater adiposity in children with detrimental consequences in adulthood [38]. Total weight gain during pregnancy is strongly associated with birth-size parameters. Birth weight of the neonatal is considered an important factor correlating with its health, but has also been recently associated with adult onset, e.g., cardiovascular disease, diabetes mellitus or even breast cancer [39]. Infants born full-term but small for gestational age (SGA) have a high risk of lifestyle diseases, whereas infants large for gestational age (LGA) have higher risk of developing high blood pressure at a young age [22]. N3 PUFA influence birth weight and gestational age, which was prolonged by 2 days and increased by 97 g in the n3 PUFA intake group (55% EPA + 37% DHA) in comparison to the control group, respectively. It was revealed in the USA that consuming more DHA capsules reduced preterm births (<34 weeks) and low-birth-weight infants (*p* = 0.0327) [22]. Reduction of preterm birth by prolonging the gestation period was also observed for women suffering from diabetes mellitus. 

A wide spectrum of multivariate methods is available in order to extract information from the data sets obtained in different nutritional and biochemical studies. Many authors have used these to distinguish the origin of food samples, to distinguish patients according to their health status, to evaluate dietary influence on microbiome, to identify the best way of feeding, or to evaluate different genomic, proteomic, lipidomic or metabolomic results [40,41,42,43,44,45]. A similar approach was applied by Aparicio et al., who applied multiple linear regression to assess the association between the socioeconomic and maternal lifestyle factors and fatty acid profile in serum of Spanish women. They showed that high educational level and older age were significantly associated with higher EPA and DHA concentrations and lower values of n6/n3 and AA/EPA. Overweight and obesity were also associated with higher values of n6/n3 ratio and AA/EPA ratio in first trimester. Hence, smoking was associated with lower DHA concentration in first trimester and higher values of n6/n3 ratio in both trimesters, which confirmed the detrimental influence of smoking for both mother and child [18]. In the present study, chemometric analyses were also successfully applied to large data sets of fatty acids profiles in serum samples of pregnant women, which allowed us to reveal subtle and discrete dependencies among serum fatty acids profiles and nutritional, anthropometric or sociodemographic factors. CA analysis of total fatty acids profile caused clear distinguishing sets of serum samples for similar features. In LDA1, 21 fatty acids quantified in serum samples have been included in the final model, which allowed three statistically significant discriminant functions. Average classification efficiency based on the calculated functions was quite high at 88.8%. The division obtained in CA resulted also from applied diet, as in the second LDA approach (LDA2) based on dietary patterns, in which 19 variables concerning diet, which were included in the final model, allowed two statistically significant functions. Average classification efficiency based on the calculated functions was slightly lower at 71.8%. This suggests, that co-existing pathological conditions can project onto the fatty acids profile as, on the one hand, this requires specific dietary modification and, on the other hand, it exerts its own influence per se. In summary, the applied LDA allowed observation of significant differences within serum samples resulting from differences in the fatty acids profile, as well as from the pathological or physiological state of the organism. 

The fatty acids profile combined with other nutritional, anthropometric and sociodemographic data composition, elaborated with various chemometric methods, can be used to provide information about dependencies in dietary and overall health of the organism, which are subtle and hidden. In the present study it was revealed by application of the chemometric approach that women in a subsequent pregnancy follow dietary recommendations and take care of their diet to a lesser extent, which results in deterioration of the fatty acids profile and higher frequency of complications. They should be given special care by a physicians, midwives and dietitians to diminish the risk of possible adverse effects affecting both mother and a child. Aydin et al. [46] found that primiparous pregnant women received more information on diet than multiparous pregnant women and made more changes in their diet. The average daily consumption of meat and meat product portions were above normal for primiparous pregnant women, but not for multiparous pregnant women, and the difference between groups was significant (*p* < 0.05). The study also found that primiparous pregnant women had higher rates of receiving information about diet than multiparous [46]. This may have been due to their excitement about pregnancy, lack of experience, or their higher levels of education. The study also found that the knowledge of pregnant women about diet during pregnancy and the dietary modifications they made during pregnancy were inadequate and ineffective especially in multiparous pregnant women [46]. However, multiparity was associated with high motivation to change diet among overweight and obese postpartum women. Approximately two thirds (68%) of participants were highly motivated to change their diet to lose weight. In the multivariable model, women with three or more children had 2.5 times the odds of high motivation compared to primigravid women [47].

Some limitations of this study should be emphasized, e.g., lack of pre-validated FFQ, which would allow assessment of the exact amounts of fatty acids intake, and lack of completed questionnaires from some (n = 26) of the investigated patients. However, application of different multivariate methods, which allowed the establishment of very interesting dependencies among examined variables and the formulation of in-depth conclusions, should be emphasized as strengths of this study. 

## 5. Conclusions

The diet of pregnant women did not fulfil all nutritional recommendations, mainly regarding dietary fat sources. Olive and rapeseed oil were the most popular edible oils. High usage of sunflower oil as well as high consumption of butter were also observed, whereas fish and fish oil intake by pregnant women was low. Application of multivariate methods of analysis allowed the exposure of various dependencies among examined variables of different types (nutritional, anthropometric, sociodemographic and biochemical). Similarity analysis revealed four clusters of patients with differing fatty acids profiles, which resulted from differences in their dietary habits. The diet of pregnant patients clearly affected the fatty acids profile in serum, which in turn had an impact on the health status of both mothers and newborns. Multiparous women followed dietary recommendations to a lesser extent, which resulted in deterioration of their fatty acids profile and higher frequency of complications. 

The observed high usage of sunflower oil is disquieting due to its lower oxidative stability, whereas high butter consumption is beneficial due to its conjugated linoleic acids supply. Pregnant women should also be encouraged to introduce fish and fish oil into their diet, as these products are rich sources of LC PUFA. Multiparous women should be given special care by medical care providers (physicians, midwifes and dietitians) along with growing attention from the government to diminish the risk of possible adverse effects affecting mother and child. The chemometric approach is a valuable tool, providing multivariate data from interdisciplinary studies’ evaluation and interpretation. 

## Figures and Tables

**Table 1:** Fatty acids profile of serum samples of pregnant patients.

		Sk1	Sk2	Sk3	Sk4	*p* Value
Cluster	Fatty Acid [%]	n = 71	n = 40	n = 11	n = 39	
Cl 1						
	c9,c12 C18:2	18.0 ± 2.4 ^a^	21.4 ± 2.3 ^a,b,c^	17.3 ± 3.8 ^b^	18.6 ± 2.0 ^c^	<0.0001
	C20:2	0.16 ± 0.03 ^a^	0.19 ± 0.04 ^a,b^	0.17 ± 0.03	0.14 ± 0.06 ^b^	<0.0001 *
Cl 2						
	C18:0	4.16 ± 0.48 ^a,b^	4.49 ± 0.36 ^a,c,d^	3.94 ± 0.8 ^c^	3.84 ± 0.49 ^b,d^	<0.0001
	c6,c9,c12 C18:3	0.18 ± 0.06 ^a,b^	0.15 ± 0.05 ^a,c^	0.42 ± 0.20 ^b,c,d^	0.15 ± 0.04 ^d^	<0.0001 *
	c5,c8,c11,c14 C20:4	3.75 ± 0.66 ^a^	4.33 ± 0.87 ^a,b^	4.14 ± 1.04	3.81 ± 0.71 ^b^	0.0010
	C22:0	0.10 ± 0.05 ^a^	0.09 ± 0.02 ^b,c^	0.09 ± 0.09 ^a,b^	0.08 ± 0.02 ^c^	0.0001 *
	C23:0	0.06 ± 0.02 ^a,b^	0.08 ± 0.05 ^a,c^	0.10 ± 0.10 ^d^	0.04 ± 0.02 ^b,c,d^	<0.0001 *
	C24:0	0.12 ± 0.03	0.13 ± 0.06	0.13 ± 0.08	0.12 ± 0.04	0.5865
Cl 3						
	C12:0	0.10 ± 0.05 ^a^	0.10 ± 0.04	0.07 ± 0.03	0.08 ± 0.02 ^a^	0.0245 *
	C14:0	1.10 ± 0.30 ^a,b^	0.83 ± 0.22 ^a^	0.88 ± 0.26	0.84 ± 0.22 ^b^	<0.0001
	C14:1	0.05 ± 0.01 ^a,b,c^	0.03 ± 0.01 ^a^	0.03 ± 0.01 ^b^	0.04 ± 0.02 ^c^	<0.0001 *
	C16:0	23.7 ± 1.9 ^a^	21.9 ± 1.3 ^a,b^	21.8 ± 1.5	23.2 ± 2.3 ^b^	<0.0001
	C16:1	2.49 ± 0.78 ^a^	1.66 ± 0.39 ^a,b^	2.11 ± 0.89	2.20 ± 0.58 ^b^	<0.0001 *
	C17:1	0.18 ± 0.05 ^a,b^	0.14 ± 0.03 ^a^	0.16 ± 0.03	0.15 ± 0.03 ^b^	<0.0001
	t9,t12 C18:2	0.08 ± 0.03 ^a,b^	0.07 ± 0.02 ^a^	0.07 ± 0.02	0.06 ± 0.02 ^b^	<0.0001
Cl 4						
	c9 C18:1	20.2 ± 2.6 ^a^	19.1 ± 2.0 ^b,c^	21.7 ± 2.8 ^b^	21.9 ± 2.0 ^a,c^	<0.0001
	c9,c12,c15 C18:3	0.59 ± 0.17 ^a^	0.57 ± 0.19 ^b^	0.92 ± 0.26 ^a,b,c^	0.56 ± 0.13 ^c^	0.0002 *
	C20:0	0.03 ± 0.03 ^a^	0.02 ± 0.01 ^b^	0.12 ± 0.11 ^a,b,c^	0.04 ± 0.05 ^c^	<0.0001 *
	C20:1	0.02 ± 0.01 ^a,b^	0.02 ± 0.01 ^c^	0.06 ± 0.02 ^a,c,d^	0.02 ± 0.01 ^b,d^	<0.0001 *
	C22:1	0.02 ± 0.01 ^a^	0.02 ± 0.01 ^b^	0.02 ± 0.00	0.03 ± 0.03 ^a,b^	0.0055 *
Cl 5						
	C15:0	0.24 ± 0.05 ^a,b^	0.21 ± 0.06 ^a^	0.21 ± 0.06	0.18 ± 0.04 ^b^	<0.0001
	C17:0	0.21 ± 0.04 ^a^	0.21 ± 0.04 ^b^	0.21 ± 0.04 ^c^	0.18 ± 0.03 ^a,b,c^	0.0002
	C20:5	0.30 ± 0.16 ^a^	0.43 ± 0.46 ^b^	0.24 ± 0.10	0.21 ± 0.08 ^a,b^	0.0011 *
	C22:6	1.77 ± 0.35 ^a^	1.86 ± 0.54 ^b^	1.80 ± 0.41	1.48 ± 0.25 ^a,b^	0.0002 *
	SFA	29.8 ± 2.2 ^a^	28.1 ± 1.6 ^a^	27.5 ± 2.2	28.6 ± 2.5	0.0001
	MUFA	23.0 ± 2.6 ^a^	20.9 ± 1.9 ^a,b,c^	24.0 ± 2.7 ^b^	24.4 ± 2.3 ^b^	<0.0001
	PUFA	24.8 ± 2.6 ^a^	28.8 ± 1.9 ^a,b,c^	25.1 ± 3.4 ^b^	25.0 ± 1.0 ^c^	<0.0001 *
	n3 PUFA	0.89 ± 0.25	1.00 ± 0.51	1.16 ± 0.32 ^a^	0.77 ± 0.15 ^a^	0.0015 *
	n6 PUFA	22.1 ± 2.5 ^a^	26.0 ± 2.0 ^a,b,c^	22.1 ± 3.2 ^b^	22.7 ± 1.9 ^c^	<0.0001
	n3 PUFA/n6 PUFA	0.04 ± 0.01 ^a^	0.04 ± 0.02 ^b^	0.05 ± 0.01 ^b,c^	0.03 ± 0.01 ^a,c^	0.0001 *

Data is presented as mean ± SD. Values with the same superscripts (letters a, b, c or d) in rows significantly differ at *p* value <0.05 in post hoc Tukey’s test or multiple comparison test (*). Sk1–Sk4—clusters of serum samples revealed in cluster analysis. Cl1–Cl5—clusters of fatty acids revealed in cluster analysis. SFA—saturated fatty acids, MUFA—monounsaturated fatty acids, PUFA—polyunsaturated fatty acids.

**Table 2:** Classification results of LDA presenting percentage predicted group membership for actual groups.

Actual Group	Correct Classification (%)	Predicted Group Membership			
		Sk1	Sk2	Sk3	Sk4
Sk1	87.3	62	6	0	3
Sk2	92.5	3	37	0	0
Sk3	100	0	0	11	0
Sk4	84.6	5	1	0	33

Sk1–Sk4—clusters of serum samples revealed in cluster analysis.

**Table 3:** Classification results of LDA presenting percentage predicted group membership for actual groups.

Actual Group	Correct Classification (%)	Predicted Group Membership			
		Sk1	Sk2	Sk3	Sk4
Sk1	80.0	48	5	2	5
Sk2	59.4	10	19	1	2
Sk3	87.5	0	0	7	1
Sk4	64.5	6	3	2	20

Sk1–Sk4—clusters of serum samples revealed in cluster analysis.

**Table 4:** Detailed characteristics of patients classified in individual clusters.

		Sk1		Sk2		Sk3		Sk4	
		n	%	n	%	n	%	n	%
diseases before pregnancy	no	57	80.3	29	72.5	9	81.8	29	74.4
	yes	14	19.7	11	27.5	2	18.2	10	25.6
	no data	0	0.0	0	0.0	0	0.0	0	0.0
diseases in pregnancy									
	no	52	73.2	26	65.0	4	36.4	21	53.8
	yes	19	26.8	14	35.0	7	63.6	18	46.2
	no data	0	0.0	0	0.0	0	0.0	0	0.0
education									
	higher	58	81.7	35	87.5	9	81.8	20	51.3
	secondary	8	11.3	3	7.5	2	18.2	14	35.9
	elementary	2	2.8	0	0.0	0	0.0	0	0.0
	vocational	0	0.0	2	5.0	0	0.0	3	7.7
	no data	3	4.2	0	0.0	0	0.0	2	5.1
BMI before pregnancy classification									
	starvation	0	0.0	0	0.0	0	0.0	0	0.0
	emaciation	3	4.2	1	2.5	0	0.0	1	2.6
	underweight	2	2.8	2	5.0	1	9.1	2	5.1
	healthy range	48	67.6	28	70.0	6	54.5	21	53.8
	overweight	13	18.3	6	15.0	3	27.3	9	23.1
	1st degree obesity	2	2.8	3	7.5	1	9.1	4	10.3
	2nd degree obesity	0	0.0	0	0.0	0	0.0	0	0.0
	3rd degree obesity	0	0.0	0	0.0	0	0.0	0	0.0
	no data	3	4.2	0	0.0	0	0.0	2	5.1
sequence number of pregnancy									
	1st	34	47.9	11	27.5	3	27.3	13	33.3
	2nd	18	25.4	15	37.5	6	54.5	12	30.8
	3rd	12	16.9	9	22.5	2	18.2	9	23.1
	4th	5	7.0	5	12.5	0	0.0	4	10.3
	5th	1	1.4	0	0.0	0	0.0	1	2.6
	6th	1	1.4	0	0.0	0	0.0	0	0.0
	no data	0	0.0	0	0.0	0	0.0	0	0.0
number of previous deliveries									
	0	36	50.7	14	35.0	4	36.4	15	38.5
	1	24	33.8	20	50.0	5	45.5	12	30.8
	2	10	14.1	3	7.5	2	18.2	9	23.1
	3	1	1.4	3	7.5	0	0.0	2	5.1
	4	0	0.0	0	0.0	0	0.0	1	2.6
	5	0	0.0	0	0.0	0	0.0	0	0.0
	no data	0	0.0	0	0.0	0	0.0	0	0.0
number of miscarriages									
	0	57	80.3	29	72.5	10	90.9	33	84.6
	1	10	14.1	9	22.5	1	9.1	5	12.8
	2	3	4.2	1	2.5	0	0.0	1	2.6
	3	1	1.4	1	2.5	0	0.0	0	0.0
	no data	0	0.0	0	0.0	0	0.0	0	0.0
tobacco smoking during pregnancy									
	no	68	95.8	40	100.0	11	100.0	37	94.9
	yes	1	1.4	0	0.0	0	0.0	0	0.0
	no data	2	2.8	0	0.0	0	0.0	2	5.1
alcohol drinking during pregnancy									
	no	68	95.8	40	0.0	11	100.0	37	94.9
	yes	0	0.0	0	0.0	0	0.0	0	0.0
	no data	3	4.2	0	0.0	0	0.0	2	5.1
delivery									
	<37th week	1	1.4	1	2.5	0	0.0	3	7.7
	≥37th week	70	98.6	39	97.5	11	100.0	36	92.3
	no data	0	0.0	0	0.0	0	0.0	0	0.0
mode of delivery									
	cesarean section	17	23.9	5	12.5	6	54.5	17	43.6
	spontaneous vaginal delivery	54	76.1	34	85.0	4	36.4	22	56.4
	vacuum-assisted vaginal delivery	0	0.0	1	2.5	1	9.1	0	0.0
	no data	0	0.0	0	0.0	0	0.0	0	0.0
gender of child									
	female	46	64.8	20	50.0	5	45.5	16	41.0
	male	25	35.2	20	50.0	6	54.5	23	59.0
	no data	0	0.0	0	0.0	0	0.0	0	0.0
Apgar scoring									
	good (8–10)	71	100.0	40	100.0	11	100.0	39	100.0
	average (4–7)	0	0.0	0	0.0	0	0.0	0	0.0
	severe (0–3)	0	0.0	0	0.0	0	0.0	0	0.0
Occurrence of developmental disorders									
	no	60	84.5	33	82.5	5	45.5	32	82.1
	yes	11	15.5	7	17.5	6	54.5	7	17.9
	no data	0	0.0	0	0.0	0	0.0	0	0.0

Sk1–Sk4—clusters of serum samples revealed in cluster analysis.

## Data Availability

The data presented in this study are available on request from the corresponding author. The data are not publicly available due to the assumption of the project, approved by the Bioethics Commission of Medical University of Warsaw (KB 158/2010 obtained 29 June 2010).

## Appendix A

**Table A1:** Characteristics of participants.

**Mother (n = 161)**		**mean** ** ± SD**	
age (years)		31.1 ± 4.5	
height (cm)		167 ± 6	
body weight before pregnancy (kg)		63.0 ± 11.8	
body weight on enrollment (kg)		77.0 ± 13.1	
BMI before pregnancy (kg/m^2^)		22.6 ± 3.8	
BMI on enrollment (kg/m^2^)		27.7 ± 4.1	
week of delivery		38.9 ± 1.2	
		n	%
diseases before pregnancy	no	124	77.0
	yes	37	23.0
	no data	0	0.0
diseases in pregnancy			
	no	103	64.0
	yes	58	36.0
	no data	0	0.0
education			
	higher	122	75.8
	secondary	27	16.8
	elementary	2	1.2
	vocational	5	3.1
	no data	5	3.1
BMI before pregnancy classification			
	starvation	0	0.0
	emaciation	5	3.1
	underweight	7	4.3
	healthy range	103	64.0
	overweight	31	19.3
	1st degree obesity	10	6.2
	2nd degree obesity	0	0.0
	3rd degree obesity	0	0.0
	no data	5	3.1
sequence number of pregnancy			
	1st	61	37.9
	2nd	51	31.7
	3rd	32	19.9
	4th	14	8.7
	5th	2	1.2
	6th	1	0.6
	no data	0	0.0
number of previous deliveries			
	0	69	42.9
	1	61	37.9
	2	24	14.9
	3	6	3.7
	4	1	0.6
	5	0	0.0
	no data	0	0.0
number of miscarriages			
	0	129	80.1
	1	25	15.5
	2	5	3.1
	3	2	1.2
	no data	0	0.0
tobacco smoking during pregnancy			
	no	156	96.9
	yes	1	0.6
	no data	4	2.5
alcohol drinking during pregnancy			
	no	156	96.9
	yes	0	0.0
	no data	5	3.1
delivery			
	<37th week	5	3.1
	≥37th week	156	96.9
	no data	0	0.0
mode of delivery			
	CC	45	28.0
	PNS	114	70.8
	VE	2	1.2
	no data	0	0.0
gender of child			
	female	87	54.0
	male	74	46.0
	no data	0	0.0
**Child (n = 161)**		**mean** **± SD**	
head circumference (cm)		34.6 ± 1.5	
chest circumference (cm)		33.7 ± 1.8	
newborn’s weight (g)		3464 ± 435	
newborn’s body length (cm)		54.5 ± 2.9	
newborn’s Ponderal Index (kg/m^3^)		21.6 ± 2.7	
Apgar 10’		9.9 ± 0.4	
		n	%
Apgar scoring			
	good (8–10)	161	100.0
	average (4–7)	0	0.0
	severe (0–3)	0	0.0
occurrence of developmental disorders			
	no	130	80.7
	yes	31	19.3
	no data	0	0.0

**Table A2:** Consumption of oils among investigated patients.

		All		Sk1		Sk2		Sk3		Sk4	
		n	%	n	%	n	%	n	%	n	%
olive											
	everyday	8	5.0	8	11.3	7	17.5	2	18.2	5	12.8
	4–6 times a week	22	13.7	3	4.2	3	7.5	0	0.0	2	5.1
	2–3 times a week	24	14.9	12	16.9	6	15.0	3	27.3	3	7.7
	once a week	18	11.2	14	19.7	1	2.5	1	9.1	2	5.1
	once a two weeks	20	12.4	10	14.1	5	12.5	1	9.1	4	10.3
	never	42	26.1	14	19.7	10	25.0	2	18.2	16	41.0
	no data	27	16.8	10	14.1	8	20.0	2	18.2	7	17.9
sunflower											
	everyday	6	3.7	3	4.2	2	5.0	0	0.0	1	2.6
	4–6 times a week	0	0.0	0	0.0	0	0.0	0	0.0	0	0.0
	2–3 times a week	19	11.8	10	14.1	4	10.0	0	0.0	5	12.8
	once a week	15	9.3	11	15.5	1	2.5	0	0.0	3	7.7
	once a two weeks	17	10.6	4	5.6	5	12.5	1	9.1	7	17.9
	never	77	47.8	33	46.5	20	50.0	8	72.7	16	41.0
	no data	27	16.8	10	14.1	8	20.0	2	18.2	7	17.9
rapeseed											
	everyday	11	6.8	1	1.4	6	15.0	0	0.0	4	10.3
	4–6 times a week	9	5.6	4	5.6	3	7.5	1	9.1	1	2.6
	2–3 times a week	18	11.2	11	15.5	3	7.5	2	18.2	2	5.1
	once a week	13	8.1	6	8.5	3	7.5	0	0.0	4	10.3
	once a two weeks	8	5.0	2	2.8	5	12.5	0	0.0	1	2.6
	never	75	46.6	37	52.1	12	30.0	6	54.5	20	51.3
	no data	27	16.8	10	14.1	8	20.0	2	18.2	7	17.9
linseed											
	everyday	1	0.6	0	0.0	1	2.5	0	0.0	0	0.0
	4–6 times a week	3	1.9	2	2.8	1	2.5	0	0.0	0	0.0
	2–3 times a week	1	0.6	0	0.0	1	2.5	0	0.0	0	0.0
	once a week	0	0.0	0	0.0	0	0.0	0	0.0	0	0.0
	once a two weeks	1	0.6	1	1.4	0	0.0	0	0.0	0	0.0
	never	128	79.5	58	81.7	29	72.5	9	81.8	32	82.1
	no data	27	16.8	10	14.1	8	20.0	2	18.2	7	17.9
corn											
	everyday	0	0.0	0	0.0	0	0.0	0	0.0	0	0.0
	4–6 times a week	0	0.0	0	0.0	0	0.0	0	0.0	0	0.0
	2–3 times a week	0	0.0	0	0.0	0	0.0	0	0.0	0	0.0
	once a week	0	0.0	0	0.0	0	0.0	0	0.0	0	0.0
	once a two weeks	0	0.0	0	0.0	0	0.0	0	0.0	0	0.0
	never	134	83.2	61	85.9	32	80.0	9	81.8	32	82.1
	no data	27	16.8	10	14.1	8	20.0	2	18.2	7	17.9
grapeseed											
	everyday	1	0.6	1	1.4	0	0.0	0	0.0	0	0.0
	4–6 times a week	1	0.6	1	1.4	0	0.0	0	0.0	0	0.0
	2–3 times a week	1	0.6	1	1.4	0	0.0	0	0.0	0	0.0
	once a week	1	0.6	1	1.4	0	0.0	0	0.0	0	0.0
	once a two weeks	2	1.2	1	1.4	1	2.5	0	0.0	0	0.0
	never	128	79.5	56	78.9	31	77.5	9	81.8	32	82.1
	no data	27	16.8	10	14.1	8	20.0	2	18.2	7	17.9
coconut											
	everyday	0	0.0	0	0.0	0	0.0	0	0.0	0	0.0
	4–6 times a week	0	0.0	0	0.0	0	0.0	0	0.0	0	0.0
	2–3 times a week	0	0.0	0	0.0	0	0.0	0	0.0	0	0.0
	once a week	0	0.0	0	0.0	0	0.0	0	0.0	0	0.0
	once a two weeks	0	0.0	0	0.0	0	0.0	0	0.0	0	0.0
	never	134	83.2	61	85.9	32	80.0	9	81.8	32	82.1
	no data	27	16.8	10	14.1	8	20.0	2	18.2	7	17.9
sesame											
	everyday	0	0.0	0	0.0	0	0.0	0	0.0	0	0.0
	4–6 times a week	0	0.0	0	0.0	0	0.0	0	0.0	0	0.0
	2–3 times a week	0	0.0	0	0.0	0	0.0	0	0.0	0	0.0
	once a week	1	0.6	0	0.0	1	2.5	0	0.0	0	0.0
	once a two weeks	0	0.0	0	0.0	0	0.0	0	0.0	0	0.0
	never	133	82.6	61	85.9	31	77.5	9	81.8	32	82.1
	no data	27	16.8	10	14.1	8	20.0	2	18.2	7	17.9
soybean											
	everyday	0	0.0	0	0.0	0	0.0	0	0.0	0	0.0
	4–6 times a week	0	0.0	0	0.0	0	0.0	0	0.0	0	0.0
	2–3 times a week	0	0.0	0	0.0	0	0.0	0	0.0	0	0.0
	once a week	1	0.6	0	0.0	1	2.5	0	0.0	0	0.0
	once a two weeks	1	0.6	0	0.0	1	2.5	0	0.0	0	0.0
	never	131	81.4	60	84.5	30	75.0	9	81.8	32	82.1
	no data	28	17.4	11	15.5	8	20.0	2	18.2	7	17.9
peanut											
	everyday	0	0.0	0	0.0	0	0.0	0	0.0	0	0.0
	4–6 times a week	0	0.0	0	0.0	0	0.0	0	0.0	0	0.0
	2–3 times a week	0	0.0	0	0.0	0	0.0	0	0.0	0	0.0
	once a week	0	0.0	0	0.0	0	0.0	0	0.0	0	0.0
	once a two weeks	0	0.0	0	0.0	0	0.0	0	0.0	0	0.0
	never	134	83.2	61	85.9	32	80.0	9	81.8	32	82.1
	no data	27	16.8	10	14.1	8	20.0	2	18.2	7	17.9
hemp											
	everyday	0	0.0	0	0.0	0	0.0	0	0.0	0	0.0
	4–6 times a week	0	0.0	0	0.0	0	0.0	0	0.0	0	0.0
	2–3 times a week	0	0.0	0	0.0	0	0.0	0	0.0	0	0.0
	once a week	0	0.0	0	0.0	0	0.0	0	0.0	0	0.0
	once a two weeks	0	0.0	0	0.0	0	0.0	0	0.0	0	0.0
	never	134	83.2	61	85.9	32	80.0	9	81.8	32	82.1
	no data	27	16.8	10	14.1	8	20.0	2	18.2	7	17.9
pumpkin											
	everyday	0	0.0	0	0.0	0	0.0	0	0.0	0	0.0
	4–6 times a week	0	0.0	0	0.0	0	0.0	0	0.0	0	0.0
	2–3 times a week	0	0.0	0	0.0	0	0.0	0	0.0	0	0.0
	once a week	0	0.0	0	0.0	0	0.0	0	0.0	0	0.0
	once a two weeks	0	0.0	0	0.0	0	0.0	0	0.0	0	0.0
	never	134	83.2	61	85.9	32	80.0	9	81.8	32	82.1
	no data	27	16.8	10	14.1	8	20.0	2	18.2	7	17.9

Sk1–Sk4—clusters of serum samples revealed in cluster analysis.

**Table A3:** Consumption of fats among investigated patients.

		All		Sk1		Sk2		Sk3		Sk4	
		n	%	n	%	n	%	n	%	n	%
margarine											
	everyday	33	20.5	11	15.5	10	25.0	2	18.2	10	25.6
	4–6 times a week	5	3.1	2	2.8	1	2.5	0	0	2	5.1
	2–3 times a week	5	3.1	2	2.8	1	2.5	0	0	2	5.1
	once a week	1	0.6	0	0	1	2.5	7	63.6	0	0
	once a two weeks	1	0.6	0	0	0	0	0	0	1	2.6
	never	89	55.3	46	64.8	19	47.5	0	0	17	43.6
	no data	27	16.8	10	14.1	8	20.0	2	18.2	7	17.9
soft margarine											
	everyday	7	4.3	3	4.2	2	5.0	0	0	2	5.1
	4–6 times a week	1	0.6	0	0	1	2.5	0	0	0	0
	2–3 times a week	1	0.6	1	1.4	0	0	0	0	0	0
	once a week	0	0	0	0	0	0	0	0	0	0
	once a two weeks	1	0.6	0	0	0	0	0	0	1	2.6
	never	124	77.0	57	80.3	29	72.5	9	81.8	29	74.4
	no data	27	16.8	10	14.1	8	20.0	2	18.2	7	17.9
butter											
	everyday	59	36.6	33	46.5	10	25.0	7	63.6	9	23.1
	4–6 times a week	13	8.1	6	8.5	3	7.5	0	0	4	10.3
	2–3 times a week	12	7.5	7	9.9	3	7.5	0	0	2	5.1
	once a week	1	0.6	0	0	1	2.5	0	0	0	0
	once a two weeks	3	1.9	2	2.8	0	0	0	0	1	2.6
	never	47	29.2	13	18.3	16	40.0	2	18.2	16	41.0
	no data	26	16.1	10	14.1	7	17.5	2	18.2	7	17.9
butter-margarine mix											
	everyday	3	1.9	2	2.8	0	0	1	9.1	0	0
	4–6 times a week	0	0	0	0	0	0	0	0	0	0
	2–3 times a week	2	1.2	1	1.4	0	0	0	0	1	2.6
	once a week	0	0	0	0	0	0	0	0	0	0
	once a two weeks	1	0.6	0	0	1	2.5	0	0	0	0
	never	128	79.5	58	81.7	31	77.5	8	72.7	31	79.5
	no data	27	16.8	10	14.1	8	20.0	2	18.2	7	17.9
lard											
	everyday	0	0.0	0	0	0	0	0	0	0	0
	4–6 times a week	0	0.0	0	0	0	0	0	0	0	0
	2–3 times a week	3	1.9	1	1.4	0	0	1	9.1	1	2.6
	once a week	0	0.0	0	0	0	0	0	0	0	0
	once a two weeks	1	0.6	0	0	1	2.5	0	0	0	0
	never	130	80.7	60	84.5	31	77.5	8	72.7	31	79.5
	no data	27	16.8	10	14.1	8	20.0	2	18.2	7	17.9
mayonnaise											
	everyday	2	1.2	2	2.8	0	0	0	0	0	0
	4–6 times a week	6	3.7	2	2.8	2	5.0	0	0	2	5.1
	2–3 times a week	10	6.2	7	9.9	3	7.5	0	0	0	0
	once a week	14	8.7	7	9.9	2	5.0	1	9.1	4	10.3
	once a two weeks	34	21.1	16	22.5	8	20.0	0	0	10	25.6
	never	68	42.2	27	38.0	17	42.5	8	72.7	16	41.0
	no data	27	16.8	10	14.1	8	20.0	2	18.2	7	17.9

Sk1–Sk4—clusters of serum samples revealed in cluster analysis.

**Table A4:** Fish consumption among investigated patients.

		All		Sk1		Sk2		Sk3		Sk4	
		n	%	n	%	n	%	n	%	n	%
salmon											
	everyday	0	0.0	0	0.0	0	0.0	0	0.0	0	0.0
	4–6 times a week	0	0.0	0	0	0	0.0	0	0.0	0	0.0
	2–3 times a week	9	5.6	4	5.6	1	2.5	0	0.0	0	0.0
	once a week	14	8.7	9	12.7	4	10.0	1	9.1	4	10.3
	once a two weeks	41	25.5	22	31.0	12	30.0	1	9.1	6	15.4
	never	71	44.1	26	36.6	16	40.0	7	63.6	22	56.4
	no data	26	16.1	10	14.1	7	17.5	2	18.2	7	17.9
tuna											
	everyday	0	0.0	0	0.0	0	0.0	0	0.0	0	0.0
	4–6 times a week	0	0.0	0	0.0	0	0.0	0	0.0	0	0.0
	2–3 times a week	4	2.5	2	2.8	0	0.0	0	0.0	1	2.6
	once a week	6	3.7	3	4.2	1	2.5	0	0.0	3	7.7
	once a two weeks	17	10.6	8	11.3	6	15.0	0	0.0	3	7.7
	never	108	67.1	48	67.6	26	65.0	9	81.8	25	64.1
	no data	26	16.1	10	14.1	7	17.5	2	18.2	7	17.9
mackerel											
	everyday	0	0.0	0	0.0	0	0.0	0	0.0	0	0.0
	4–6 times a week	0	0.0	0	0.0	0	0.0	0	0.0	0	0.0
	2–3 times a week	2	1.2	1	1.4	0	0.0	0	0.0	1	2.6
	once a week	7	4.3	3	4.2	0	0.0	0	0.0	3	7.7
	once a two weeks	37	23.0	16	22.5	13	32.5	1	9.1	8	20.5
	never	89	55.3	41	57.7	20	50.0	8	72.7	20	51.3
	no data	26	16.1	10	14.1	7	17.5	2	18.2	7	17.9
trout											
	everyday	0	0.0	0	0.0	0	0.0	0	0.0	0	0.0
	4–6 times a week	0	0.0	0	0.0	0	0.0	0	0.0	0	0.0
	2–3 times a week	0	0.0	0	0.0	0	0.0	0	0.0	0	0.0
	once a week	3	1.9	2	2.8	0	0.0	0	0.0	1	2.6
	once a two weeks	13	8.1	7	9.9	3	7.5	1	9.1	2	5.1
	never	119	73.9	52	73.2	30	75.0	8	72.7	29	74.4
	no data	26	16.1	10	14.1	7	17.5	2	18.2	7	17.9
cod											
	everyday	0	0.0	0	0.0	0	0.0	0	0.0	0	0.0
	4–6 times a week	0	0.0	0	0.0	0	0.0	0	0.0	0	0.0
	2–3 times a week	1	0.6	0	0.0	1	2.5	0	0.0	0	0.0
	once a week	8	5.0	4	5.6	1	2.5	2	18.2	1	2.6
	once a two weeks	33	20.5	17	23.9	7	17.5	1	9.1	8	20.5
	never	93	57.8	40	56.3	24	60.0	6	54.5	23	59.0
	no data	26	16.1	10	14.1	7	17.5	2	18.2	7	17.9
herring											
	everyday	0	0.0	0	0.0	0	0.0	0	0.0	0	0.0
	4–6 times a week	2	1.2	0	0.0	1	2.5	0	0.0	1	2.6
	2–3 times a week	4	2.5	0	0.0	2	5.0	0	0.0	1	2.6
	once a week	7	4.3	3	4.2	1	2.5	1	9.1	3	7.7
	once a two weeks	25	15.5	17	23.9	3	7.5	1	9.1	5	12.8
	never	97	60.2	41	57.7	26	65.0	7	63.6	23	59.0
	no data	26	16.1	10	14.1	7	17.5	2	18.2	7	17.9
sardine											
	everyday	0	0	0	0	0	0	0	0	0	0
	4–6 times a week	0	0	0	0	0	0	0	0	0	0
	2–3 times a week	0	0	0	0	0	0	0	0	0	0
	once a week	0	0	0	0	0	0	0	0	0	0
	once a two weeks	1	0.6	1	1.4	0	0	0	0	0	0
	never	134	83.2	60	84.5	33	82.5	9	81.8	32	82.1
	no data	26	16.1	10	14.1	7	17.5	2	18.2	7	17.9
eel											
	everyday	0	0	0	0	0	0	0	0	0	0
	4–6 times a week	0	0	0	0	0	0	0	0	0	0
	2–3 times a week	0	0	0	0	0	0	0	0	0	0
	once a week	0	0	0	0	0	0	0	0	0	0
	once a two weeks	0	0	0	0	0	0	0	0	0	0
	never	135	83.9	61	85.9	33	82.5	9	81.8	32	82.1
	no data	26	16.1	10	14.1	7	17.5	2	18.2	7	17.9
halibut											
	everyday	0	0	0	0	0	0	0	0	0	0
	4–6 times a week	0	0	0	0	0	0	0	0	0	0
	2–3 times a week	0	0	0	0	0	0	0	0	0	0
	once a week	0	0	0	0	0	0	0	0	0	0
	once a two weeks	3	1.9	1	1.4	2	5.0	0	0	0	0
	never	132	82.0	60	84.5	31	77.5	9	81.8	32	82.1
	no data	26	16.1	10	14.1	7	17.5	2	18.2	7	17.9
sprat											
	everyday	0	0	0	0	0	0	0	0	0	0
	4–6 times a week	0	0	0	0	0	0	0	0	0	0
	2–3 times a week	0	0	0	0	0	0	0	0	0	0
	once a week	2	1.2	1	1.4	1	2.5	0	0	0	0
	once a two weeks	6	3.7	3	4.2	1	2.5	0	0	2	5.1
	never	127	78.9	57	80.3	31	77.5	9	81.8	30	76.9
	no data	26	16.1	10	14.1	7	17.5	2	18.2	7	17.9
pollock											
	everyday	0	0	0	0	0	0	0	0	0	0
	4–6 times a week	0	0	0	0	0	0	0	0	0	0
	2–3 times a week	0	0	0	0	0	0	0	0	0	0
	once a week	1	0.6	0	0	0	0	0	0	0	0
	once a two weeks	2	1.2	1	1.4	1	2.5	0	0	1	2.6
	never	132	82.0	60	84.5	32	80.0	9	81.8	31	79.5
	no data	26	16.1	10	14.1	7	17.5	2	18.2	7	17.9

Sk1–Sk4—clusters of serum samples revealed in cluster analysis.

**Table A5:** Consumption of nuts among investigated patients.

		All		Sk1		Sk2		Sk3		Sk4	
		n	%	n	%	n	%	n	%	n	%
hazelnuts											
	everyday	2	1.2	1	1.4	1	2.5	0	0.0	0	0.0
	4–6 times a week	3	1.9	1	1.4	2	5.0	0	0.0	0	0.0
	2–3 times a week	9	5.6	2	2.8	3	7.5	1	9.1	3	7.7
	once a week	8	5.0	2	2.8	3	7.5	1	9.1	2	5.1
	once a two weeks	29	18.0	16	22.5	7	17.5	1	9.1	5	12.8
	never	83	51.6	39	54.9	16	40.0	6	54.5	22	56.4
	no data	27	16.8	10	14.1	8	20.0	2	18.2	7	17.9
walnuts											
	everyday	0	0.0	0.0	0.0	4	10.0	0.0	0.0	1	2.6
	4–6 times a week	5	3.1	0.0	0.0	0.0	0.0	0.0	0.0	0.0	0.0
	2–3 times a week	9	5.6	2	2.8	4	10.0	1	9.1	2	5.1
	once a week	10	6.2	2	2.8	1	2.5	2	18.2	5	12.8
	once a two weeks	33	20.5	19	26.8	8	20.0	1	9.1	5	12.8
	never	77	47.8	38	53.5	15	37.5	5	45.5	19	48.7
	no data	27	16.8	10	14.1	8	20.0	2	18.2	7	17.9
cashews											
	everyday	0	0	0	0.0	0	0.0	0	0.0	0	0.0
	4–6 times a week	0	0	0	0.0	0	0.0	0	0.0	0	0.0
	2–3 times a week	7	4.3	2	2.8	4	10.0	0	0.0	1	2.6
	once a week	3	1.9	1	1.4	0	0.0	1	9.1	1	2.6
	once a two weeks	15	9.3	5	7.0	4	10.0	1	9.1	5	12.8
	never	109	67.7	53	74.6	24	60.0	7	63.6	25	64.1
	no data	27	16.8	10	14.1	8	20.0	2	18.2	7	17.9
pistachios											
	everyday	0	0	0	0.0	0	0.0	0	0.0	0	0.0
	4–6 times a week	0	0	0	0.0	0	0.0	0	0.0	0	0.0
	2–3 times a week	3	1.9	0	0.0	2	5.0	1	9.1	1	2.6
	once a week	5	3.1	3	4.2	0	0.0	0	0.0	1	2.6
	once a two weeks	13	8.1	6	8.5	3	7.5	0	0.0	4	10.3
	never	113	70.2	52	73.2	27	67.5	8	72.7	26	66.7
	no data	27	16.8	10	14.1	8	20.0	2	18.2	7	17.9
almonds											
	everyday	6	3.7	1	1.4	4	10.0	0	0.0	1	2.6
	4–6 times a week	3	1.9	0	0.0	2	5.0	1	9.1	0	0.0
	2–3 times a week	8	5.0	2	2.8	3	7.5	1	9.1	2	5.1
	once a week	8	5.0	4	5.6	0	0.0	1	9.1	3	7.7
	once a two weeks	26	16.1	15	21.1	8	20.0	2	18.2	1	2.6
	never	83	51.6	39	54.9	15	37.5	4	36.4	25	64.1
	no data	27	16.8	10	14.1	8	20.0	2	18.2	7	17.9
peanuts											
	everyday	1	0.6	1	1.4	0	0.0	0	0.0	0	0.0
	4–6 times a week	2	1.2	0	0.0	2	5.0	0	0.0	0	0.0
	2–3 times a week	2	1.2	1	1.4	0	0.0	0	0.0	1	2.6
	once a week	3	1.9	1	1.4	0	0.0	1	9.1	1	2.6
	once a two weeks	6	3.7	4	5.6	2	5.0	0	0.0	0	0.0
	never	120	74.5	54	76.1	28	70.0	8	72.7	30	76.9
	no data	27	16.8	10	14.1	8	20.0	2	18.2	7	17.9

Sk1–Sk4—clusters of serum samples revealed in cluster analysis.

**Table A6:** Consumption of eggs among investigated patients.

		All		Sk1		Sk2		Sk3		Sk4	
		n	%	n	%	n	%	n	%	n	%
eggs											
	everyday	11	6.8	5	7.0	4	10.0	0	0.0	2	5.1
	4–6 times a week	2	1.2	1	1.4	1	2.5	0	0.0	0	0.0
	2–3 times a week	47	29.2	22	31.0	9	22.5	3	27.3	13	33.3
	once a week	55	34.2	25	35.2	16	40.0	5	45.5	9	23.1
	once a two weeks	14	8.7	5	7.0	2	5.0	0	0.0	7	17.9
	never	6	3.7	3	4.2	1	2.5	1	9.1	1	2.6
	no data	26	16.1	10	14.1	7	17.5	2	18.2	7	17.9

Sk1–Sk4—clusters of serum samples revealed in cluster analysis.

**Table A7:** Flakes, cereals and seeds consumption among investigated patients.

		All		Sk1		Sk2		Sk3		Sk4	
		n	%	n	%	n	%	n	%	n	%
oatmeal											
	everyday	7	4.3	2	2.8	2	5.0	1	9.1	2	5.1
	4–6 times a week	10	6.2	6	8.5	1	2.5	0	0.0	3	7.7
	2–3 times a week	21	13.0	10	14.1	4	10.0	3	27.3	4	10.3
	once a week	22	13.7	9	12.7	7	17.5	2	18.2	4	10.3
	once a two weeks	9	5.6	3	4.2	3	7.5	0	0.0	3	7.7
	never	65	40.4	31	43.7	15	37.5	3	27.3	16	41.0
	no data	27	16.8	10	14.1	8	20.0	2	18.2	7	17.9
cornflakes											
	everyday	9	5.6	6	8.5	0	0.0	2	18.2	1	2.6
	4–6 times a week	4	2.5	3	4.2	0	0.0	1	9.1	0	0.0
	2–3 times a week	13	8.1	9	12.7	1	2.5	0	0.0	3	7.7
	once a week	6	3.7	2	2.8	0	0.0	1	9.1	3	7.7
	once a two weeks	3	1.9	1	1.4	1	2.5	1	9.1	0	0.0
	never	99	61.5	40	56.3	30	75.0	4	36.4	25	64.1
	no data	27	16.8	10	14.1	8	20.0	2	18.2	7	17.9
rice flakes											
	everyday	0	0.0	0	0.0	0	0.0	0	0.0	0	0.0
	4–6 times a week	0	0.0	0	0.0	0	0.0	0	0.0	0	0.0
	2–3 times a week	1	0.6	0	0.0	1	2.5	0	0.0	0	0.0
	once a week	2	1.2	1	1.4	0	0.0	0	0.0	1	2.6
	once a two weeks	0	0	0	0	0	0	0	0	0	0
	never	131	81.4	60	84.5	31	77.5	9	81.8	31	79.5
	no data	27	16.8	10	14.1	8	20.0	2	18.2	7	17.9
sunflower seeds											
	everyday	4	2.5	1	1.4	3	7.5	0	0.0	0	0.0
	4–6 times a week	3	1.9	0	0.0	3	7.5	0	0.0	0	0.0
	2–3 times a week	4	2.5	2	2.8	0	0.0	1	9.1	1	2.6
	once a week	10	6.2	5	7.0	1	2.5	1	9.1	3	7.7
	once a two weeks	13	8.1	6	8.5	5	12.5	0	0.0	2	5.1
	never	100	62.1	47	66.2	20	50.0	7	63.6	26	66.7
	no data	27	16.8	10	14.1	8	20.0	2	18.2	7	17.9
pumpkin seeds											
	everyday	2	1.2	0	0.0	2	5.0	0	0.0	0	0.0
	4–6 times a week	2	1.2	1	1.4	1	2.5	0	0.0	0	0.0
	2–3 times a week	1	0.6	1	1.4	0	0.0	0	0.0	0	0.0
	once a week	7	4.3	3	4.2	1	2.5	1	9.1	2	5.1
	once a two weeks	8	5.0	3	4.2	4	10.0	0	0.0	1	2.6
	never	114	70.8	53	74.6	24	60.0	8	72.7	29	74.4
	no data	27	16.8	10	14.1	8	20.0	2	18.2	7	17.9
wheat bread											
	everyday	11	6.8	3	4.2	1	2.5	5	45.5	2	5.1
	4–6 times a week	2	1.2	2	2.8	0	0.0	0	0.0	0	0.0
	2–3 times a week	2	1.2	1	1.4	1	2.5	0	0.0	0	0.0
	once a week	0	0.0	0	0.0	0	0.0	0	0.0	0	0.0
	once a two weeks	2	1.2	1	1.4	1	2.5	0	0.0	0	0.0
	never	116	72.0	54	76.1	29	72.5	4	36.4	29	74.4
	no data	28	17.4	10	14.1	8	20.0	2	18.2	8	20.5
rye bread											
	everyday	39	24.2	19	26.8	12	30.0	1	9.1	7	17.9
	4–6 times a week	10	6.2	4	5.6	5	12.5	0	0.0	1	2.6
	2–3 times a week	4	2.5	1	1.4	2	5.0	0	0.0	1	2.6
	once a week	3	1.9	2	2.8	0	0.0	1	9.1	0	0.0
	once a two weeks	2	1.2	1	1.4	1	2.5	0	0.0	0	0.0
	never	76	47.2	34	47.9	12	30.0	7	63.6	23	59.0
	no data	27	16.8	10	14.1	8	20.0	2	18.2	7	17.9
whole wheat bread											
	everyday	32	19.9	15	21.1	5	12.5	1	9.1	11	28.2
	4–6 times a week	16	9.9	6	8.5	5	12.5	0	0.0	5	12.8
	2–3 times a week	9	5.6	4	5.6	1	2.5	0	0.0	4	10.3
	once a week	8	5.0	6	8.5	1	2.5	0	0.0	1	2.6
	once a two weeks	4	2.5	2	2.8	0	0.0	0	0.0	2	5.1
	never	64	39.8	28	39.4	20	50.0	7	63.6	9	23.1
	no data	28	17.4	10	14.1	8	20.0	3	27.3	7	17.9

Sk1–Sk4—clusters of serum samples revealed in cluster analysis.

**Table A8:** Selected food products consumption and diet supplementation among investigated patients.

		All		Sk1		Sk2		Sk3		Sk4	
		n	%	n	%	n	%	n	%	n	%
avocado											
	everyday	1	0.6	1	1.4	0	0.0	0	0.0	0	0.0
	4–6 times a week	0	0.0	0	0.0	0	0.0	0	0.0	0	0.0
	2–3 times a week	2	1.2	1	1.4	0	0.0	1	9.1	0	0.0
	once a week	6	3.7	3	4.2	1	2.5	0	0.0	2	5.1
	once a two weeks	7	4.3	3	4.2	3	7.5	0	0.0	1	2.6
	never	118	73.3	53	74.6	28	70.0	8	72.7	29	74.4
	no data	27	16.8	10	14.1	8	20.0	2	18.2	7	17.9
lettuce											
	everyday	15	9.3	8	11.3	4	10.0	0	0.0	3	7.7
	4–6 times a week	2	1.2	0	0.0	1	2.5	0	0.0	1	2.6
	2–3 times a week	32	19.9	14	19.7	6	15.0	5	45.5	7	17.9
	once a week	23	14.3	11	15.5	6	15.0	0	0.0	6	15.4
	once a two weeks	17	10.6	8	11.3	5	12.5	0	0.0	4	10.3
	never	45	28.0	20	28.2	10	25.0	4	36.4	11	28.2
	no data	27	16.8	10	14.1	8	20.0	2	18.2	7	17.9
spinach											
	everyday	0	0.0	0	0.0	0	0.0	0	0.0	0	0.0
	4–6 times a week	1	0.6	1	1.4	0	0.0	0	0.0	0	0.0
	2–3 times a week	7	4.3	6	8.5	1	2.5	0	0.0	0	0.0
	once a week	20	12.4	7	9.9	8	20.0	1	9.1	4	10.3
	once a two weeks	24	14.9	12	16.9	5	12.5	2	18.2	5	12.8
	never	82	50.9	35	49.3	18	45.0	6	54.5	23	59.0
	no data	27	16.8	10	14.1	8	20.0	2	18.2	7	17.9
tofu											
	everyday	0	0.0	0	0.0	0	0.0	0	0.0	0	0.0
	4–6 times a week	0	0.0	0	0.0	0	0.0	0	0.0	0	0.0
	2–3 times a week	1	0.6	0	0.0	0	0.0	0	0.0	1	2.6
	once a week	0	0.0	0	0.0	0	0.0	0	0.0	0	0.0
	once a two weeks	1	0.6	1	1.4	0	0.0	0	0.0	0	0.0
	never	132	82.0	60	84.5	32	80.0	9	81.8	31	79.5
	no data	27	16.8	10	14.1	8	20.0	2	18.2	7	17.9
fish oil											
	yes	5	3.1	3	4.2	1	2.5	0	0.0	1	2.6
	no	151	93.8	65	91.5	39	97.5	11	100.0	36	92.3
	no data	5	3.1	3	4.2	0	0.0	0	0.0	2	5.1
vitamin supplementation											
	yes	100	62.1	47	66.2	24	60.0	8	72.7	21	53.8
	no	56	34.8	21	29.6	16	40.0	3	27.3	16	41.0
	no data	5	3.1	3	4.2	0	0.0	0	0.0	2	5.1
omega supplements											
	yes	65	40.4	28	39.4	18	45.0	8	72.7	11	28.2
	no	90	55.9	39	54.9	22	55.0	3	27.3	26	66.7
	no data	6	3.7	4	5.6	0	0.0	0	0.0	2	5.1
potato fries											
	yes	50	31.1	28	39.4	11	27.5	1	9.1	10	25.6
	no	93	57.8	36	50.7	23	57.5	8	72.7	26	66.7
	no data	18	11.2	7	9.9	6	15.0	2	18.2	3	7.7
chips											
	yes	30	18.6	19	26.8	6	15.0	1	9.1	4	10.3
	no	113	70.2	45	63.4	28	70.0	8	72.7	32	82.1
	no data	18	11.2	7	9.9	6	15.0	2	18.2	3	7.7

Sk1–Sk4—clusters of serum samples revealed in cluster analysis.

**Table A9:** Coefficients and average value of canonical variables included in the final model (LDA1).

**Coefficients of Canonical Variables**			
**Variable** **(Discriminatory Power)**	**DF1** **(62.4%)**	**DF2** **(23.1%)**	**DF3** **(14.5%)**
C20:1	1.019033	0.153469	−0.384475
C17:1	−0.197239	−0.335925	0.670768
c6,c9,c12 C18:3	1.226704	0.142788	0.518516
C18:0	−0.353679	−0.164360	−0.235229
C22:1	−0.368181	0.525440	−0.195506
c9 C18:1	−0.115208	0.588598	0.157178
C23:0	0.165942	−0.555285	−0.060737
C16:1	−0.606104	0.433519	−0.186427
c5c8c11c14c17 C20:5	−0.533300	−0.198048	0.231662
c9,c12,c15 C18:3	0.592131	0.026560	−0.305218
C15:0	0.162175	−0.212680	0.677425
C20:2	0.251177	−0.482919	0.194679
c5,c8,c11,c14 C20:4	−0.108988	0.130942	−0.510834
c9,c12 C18:2	−0.496911	−0.116980	−0.288841
C22:0	−0.206239	0.064151	0.235650
C12:0	−0.141885	−0.254590	−0.070607
C17:0	−0.193858	−0.144524	−0.347023
C24:0	−0.025364	−0.031390	−0.354546
c4,c7,c10,c13,c16,c19 C22:6	0.006504	−0.306643	0.109614
C20:0	0.185902	−0.244927	−0.152906
C14:0	−0.262623	0.241964	0.232538
**Average Value of Canonical Variables**			
Sk1	−0.245045	−0.21546	1.05831
Sk2	−0.856846	−1.48900	−1.10289
Sk3	7.274431	−0.00424	−0.47649
Sk4	−0.726839	1.92062	−0.66112

Sk1–Sk4—clusters of serum samples revealed in cluster analysis.

**Table A10:** Coefficients and average value of canonical variables included in the final model (LDA2).

**Coefficients of Canonical Variables**		
**Variable** **(Discriminatory Power)**	**DF1** **(49.7%)**	**DF2** **(34.1%)**
Wheat bread	−0.534250	0.172987
Almonds	−0.174052	0.299568
Butter	−0.156545	−0.228098
Whole wheat bread	0.193427	0.022746
Butter-margarine mix	−0.549327	−0.169966
Omega-3 supplementation	−0.956393	0.184221
Cornflakes	−0.076700	−0.208335
Rye bread	0.149223	0.092971
Grapeseed oil	−0.021209	−0.681737
Chips	0.270719	−0.861812
Salmon	0.033694	−0.537936
Lard	0.148335	0.932102
Sunflower oil	0.172069	−0.308455
Walnuts	0.157714	0.360171
Vitamin supplementation	0.071907	−0.809480
Tofu	0.770064	0.321142
Cod	−0.587247	−0.130541
Herring	−0.411779	0.068285
Halibut	2.197515	0.803059
constant	−0.009766	2.099391
**Average Value of Canonical Variables**		
Sk1	−0.07187	−0.791351
Sk2	0.38672	0.792241
Sk3	−3.27057	0.829717
Sk4	0.58161	0.474202

Sk1–Sk4—clusters of serum samples revealed in cluster analysis.

## Appendix B

**Pregnant Patient Questionnaire** date .....................

**General interview** (**filled in by medical staff**)**:**

(1) Name and surname: ............. number of medical history: ............. ID ............
(2) Age: .............
(3) Education/profession: .............
(4) Body mass before pregnancy ............. present body mass: ............. height............... BMI: .............
(5) Number of current pregnancy: .............
(6) previous obstetric interview (number of deliveries: ............., number of miscarriages.............)
(7) chronic diseases before pregnancy (diseases of the kidneys, liver, thyroid, etc.): .............
(8) Does the family (parents, siblings, grandparents) have liver diseases, diabetes, arterial hypertension? ..............................................................................

**Current pregnancy interview (filled in by medical staff):**

(9) date of the last menstrual period: ............. date of labour: ...........
  Diseases in current pregnancy:
(10) hypertension.............
(11) gestational diabetes .............
(12) cholestasis .............
(13) Other diseases complicating pregnancy? .............
(14) How long/from which week of pregnancy? .............
(15) Do you smoke cigarettes when you are pregnant? ............How many/24 h: .............
(16) Do you drink alcohol when you are pregnant? ..............How much? ............How often? .............

**Data on child** (**filled in by medical staff**)**:**

Data of labour/Birth date: .............

Wee of pregnancy: .............

Way of delivery: .............

Indications for a surgical delivery: .............

Sex of the child: .............

Body mass: ............. Length: ............. Ponderal Index: .............

Apgar scoring after 1′............. 5′............. 10′.............

Umbilical pH............. lactates: .............

Are there any abnormalities in the child? .............

**Food Frequency Questionnaire (filled in by the Patient)**

**Fish**								
**Food Product**	**How Often Consumed in Pregnancy?**						**Amount per Serving (Grams)**	**Number of Serving per Week**
	**Never**	**Once a Two Weeks**	**ONCE A WEEK**	**2–3 Times a Week**	**4–6 Times a Week**	**Everyday**		
salmon								
tuna								
mackerel								
trout								
cod								
herring								
sardine								
eel								
halibut								
sprat								
other								

**Sea food**								
**Food Product**	**How Often Consumed in Pregnancy?**						**Amount per Serving (Grams)**	**Number of Serving per Week**
	**Never**	**Once a Two Weeks**	**Once a Week**	**2–3 Times a Week**	**4–6 Times a Week**	**Everyday**		
clams								
prawns								
oysters								
mixed shells								
squids								
lobsters								
other								

**Nuts**								
**Food Product**	**How Often Consumed in Pregnancy?**						**Amount per Serving (Grams)**	**Number of Serving per Week**
	**Never**	**Once a Two Weeks**	**Once a Week**	**2–3 Times a Week**	**4–6 Times a Week**	**Everyday**		
hazelnuts								
walnuts								
cashews								
pistachios								
almonds								
peanuts								
other								

**Edible oils, fats**								
**Food Product**	**How Often Consumed in Pregnancy?**						**Amount per Serving (Tablespoons)**	**Number of Serving per Week**
	**Never**	**Once a Two Weeks**	**Once a Week**	**2–3 Times a Week**	**4–6 Times a Week**	**Everyday**		
olive								
sunflower								
rapeseed								
linseed								
corn								
grapeseed								
coconut								
sesame								
soybean								
peanut								
hemp								
pumpkin								
other								

**Food Product**	**How Often Consumed in Pregnancy?**						**Amount per Serving (Tablespoons)**	**Number of Serving per Week**
	**Never**	**Once a two Weeks**	**Once a Week**	**2–3 Times a Week**	**4–6 Times a Week**	**Everyday**		
margarine								
soft margarine								
butter								
butter-margarine mix								
lard								
mayonnaise								

**Eggs**								
**Food Product**	**How Often Consumed in Pregnancy?**						**How Many Pieces per Serving**	**Number of Serving per Week**
	**Never**	**Once a Two Weeks**	**Once a Week**	**2–3 Times a Week**	**4–6 Times a Week**	**Everyday**		
eggs								

**Flakes, cereals and seeds, fruits, vegetables**								
**Food Product**	**How Often Consumed in Pregnancy?**						**Amount per Serving (Tablespoons/Slices)**	**Number of Serving per Week**
	**Never**	**Once a Two Weeks**	**Once a Week**	**2–3 Times a Week**	**4–6 Times a Week**	**Everyday**		
oatmeal								
cornflakes								
rice flakes								
sunflower seeds								
pumpkin seeds								
wheat bread								
rye bread								
whole wheat bread								
other								

**Food Product**	**How Often Consumed in Pregnancy?**						**Amount per Serving (Grams)**	**Number of Serving per Week**
	**Never**	**Once a Two Weeks**	**Once a week**	**2–3 Times a Week**	**4–6 Times a Week**	**Everyday**		
avocado								
lettuce								
spinach								
tofu								
other								

(1) Do you drink fish oil? yes no
  How many tablespoons per serving and how often? .............................................................................................................................
  Have you drunk fish oil in the last 7 days?
  ..............................................................................................................................
(2) Do you use vitamin supplementation during pregnancy? Please enter the exact name and dose.
  ........................................................................................................................................................................................................................................................................................................................................................................................
(3) Do you use omega supplementation during pregnancy? Please enter the exact name and dose .............................................................................................................................................................................................................................................................................................................................................................................................................................
(4) Please list the names of all drugs (and in what dose) you were/are taking during pregnancy?
  .....................................................................................................................................................................................................................................................................................................................................................................................................................................................................................................................................................................................................................................................
(5) Did you eat potato fries during pregnancy? yes no
(6) Did you eat chips during pregnancy? yes no

Thank you very much for completing the survey!
